# Positive and negative impacts of electrical infrastructure on animal biodiversity: A systematic review

**DOI:** 10.1007/s00442-025-05780-7

**Published:** 2025-08-13

**Authors:** Adam J. Bennett, David M. Watson, Maggie J. Watson

**Affiliations:** 1 Charles Sturt University, 386 Elizabeth Mitchell Drive, Thurgoona, NSW 2640 Australia; 2 Bird Group, The Natural History Museum, Tring, Hertfordshire HP23 6AP UK

**Keywords:** Collision, Electrical Infrastructure, Electrocution, Habitat Alteration, Mitigation

## Abstract

Electrical infrastructure networks are predicted to greatly expand in response to the expected 48% increase in global energy demand by 2040. While assessments of electrical infrastructure are abundant, mixed effects on animal diversity have been reported, warranting further evaluation to determine true effects. We conducted a systematic literature review covering individual, population, and community-level assessments to determine the impacts posed to animal biodiversity by electrical infrastructure and the measures to reduce impacts. Our results show that the literature around electrical infrastructure and animal biodiversity has grown exponentially since the start of the millennium and is biased towards birds and mammals in economically developed countries. We found the majority of reported impacts were negative, occurring primarily through barrier effects (collision) and use of linear features as a resource (electrocution), causing significant population impacts in select species. However, we also found that electrical infrastructure provided positive effects (increased habitat provisioning) to certain taxonomic groups (e.g. corvids, storks), which suggests the potential to encourage conservation efforts using appropriate mitigation on electrical infrastructure. We found the majority of current literature detailed mitigation measures focused on reducing collisions through line (51%) and tower alterations (30%), but often omitted the impacts of EMF, noise, and UV, indicating significant knowledge gaps. On a wider scale, we found that mortality from electrical infrastructure ranked lower than building collisions and predation from feral cats, but remained higher than other anthropogenic energy sources, indicating the potential to cause significant impacts to large-bodied bird species if not addressed.

**Supplementary Information:**

The online version contains supplementary material available at 10.1007/s00442-025-05780-7.

## Introduction

Natural systems characterised by high levels of biodiversity, provide substantial benefits to humans through ecosystem services, including but not limited to oxygen production, pollination services, nutrient recycling, and water regulation. Recent estimates show that 60% of global ecosystem services are now depleted, with the greatest losses occurring within the last 50 years (Mooney et al. 2009). The International Union for Conservation of Nature (IUCN) now predicts that a minimum of 10,967 species are at risk of extinction and has already documented 38 species extinctions to be directly caused by anthropogenic induced climate change. Furthermore, the global energy demand is expected to increase by a rate of 48% by 2040, with 80% supplied by fossil fuels. Conservation ecologists are now tasked with stemming the rate of species losses and maintaining the critical ecosystem services they provide, intertwining both biodiversity and climate change at the precipice of current scientific research (Bellard et al. 2012; Moodley and Trois 2021).

At present, the most pragmatic method for achieving climate change and biodiversity targets is to accelerate the development towards renewable energy. This requires using strategies such as solar and wind and the development of electrical infrastructure linking renewables to national grids through associated power/transmission lines. However, as humanity transitions into this new phase of energy production, it is vital to develop sustainable practices without incurring additional species losses in addition to those already posed by climate change and other degradative practices such as habitat alteration and destruction (Martin et al. 2022).

Generally, power plants are situated long distances away from major consumption areas resulting in large expanses and networks of transmission lines (Biasotto and Kindel 2018). Furthermore, poorly managed electrical infrastructure can impose a variety of detrimental impacts that can impact animal biodiversity through direct mortality by collision and electrocution, habitat alteration and degradation from invasive species. Functional trait assessments have already identified several orders (hawks and eagles (Accipitriformes), seriemas (Cariamiformes), New World vultures (Cathartiformes), herons and storks (Ciconiformes), falcons (Falconiformes), bustards and allies (Otidiformes) and owls (Strigiformes)) to be at increased risk due to larger body masses, larger wingspans, and life-history traits such as small clutch sizes (Thaxter et al. 2017, Wang et al. 2018, Buechley et al. 2019). Furthermore, interactions with electrical infrastructure can lead to infrastructure damage, fire, and power outages, indicating that it is in the best interest of energy providers to mitigate adverse effects and encourage species conservation (Martin et al. 2022).

On the other hand, electrical infrastructure has the potential to induce positive effects on animal biodiversity by increasing hunting efficacy of predatory species such as the saker falcon (*Falco cherrug*) and nesting success of white stork (*Ciconia ciconia*) (Puzovic 2008; Vaitkuviene and Dagys 2015). Certain modifications, such as insulators and additions, such as platforms, alongside clearing management strategies that focus on maintaining habitat diversity through the creation of gradual ecotones can further offset detrimental effects, enhance, and maintain local mammal biodiversity (Clarke et al. 2006, Salek et al. 2020; Hrouda and Brlik 2021).

Environmental Impact Assessments (EIAs) need to focus on determining whether the negative impacts of electrical infrastructure outweigh the positive impacts and if existing mitigation measures are adequate to offset the detrimental effects posed to animal biodiversity (Clarke et al. 2006). Counterintuitively, more economically developed countries (MEDCs) such as France, Germany, New Zealand, and Sweden are currently considered data poor in their understanding of the impacts of electrical infrastructure. With powerlines alone now increasing at a rate of 5% per year, with an expected 80,000,000 km^2^ of additions by 2040 to existing electrical grids to match the increasing global demand for energy, there is now immediate urgency for detailed systematic assessments (Jenkins et al. 2010; Alcayde-Garcia et al. 2022; IEA 2023).

In this paper, we conducted a systematic review (aggregative full literature review) to search and synthesise the current nature and extent of knowledge around interactions between electrical infrastructure and animal biodiversity. Specifically, we attempt to address the following questions regarding electrical infrastructure: (1) How are powerlines and associated electrical infrastructure impacting animal biodiversity? (2) Are the reported impacts from electrical infrastructure considered positive or negative? (3) What are the main mitigation measures in place on electrical infrastructure? (4) Does mortality from powerlines and electrical infrastructure significantly impact populations in comparison to other sources of anthropogenic mortality (e.g. building collisions and predation by domestic or feral cats (*Felis catus*)?

## Methods

An initial search of Google Scholar was used to identify and collect data on studies associated with animal biodiversity and powerline interactions using a search of the following terms: “powerlines AND biodiversity,” “powerlines AND biodiversity mitigation,” “powerlines AND birds,” “powerlines AND mammals” and “powerlines AND reptiles.” Publications pertaining to plants were excluded (Table 1). Initial searches were conducted on Google Scholar, and then the same searches were conducted across BioOne, EBSCOHOST (Greenfile), SCOPUS, Nature, and Web of Science databases. Review papers were included to help identify existing knowledge gaps and further clarify research objectives. A secondary search of the grey literature (interview statements, news articles, policy statements, technical reports, and website articles obtained from Google searches) was then incorporated to further increase data availability from non-academic sources (Haddaway et al. 2015).

**Table 1:** Search terms used for identifying primary and grey literature around electrical infrastructure impacts

Primary search term	Secondary search term
Anthropogenic Infrastructure	Biodiversity
	Biodiversity Mitigation
	Birds
	Collisions
	Electrocution
	Impacts
	Mammals
	Reptiles
	Rights of Way
Electrical Infrastructure	Biodiversity
	Biodiversity Mitigation
	Birds
	Collisions
	Electrocution
	Impacts
	Mammals
	Reptiles
	Rights of Way
Powerlines	Biodiversity
	Biodiversity Mitigation
	Birds
	Collisions
	Electrocution
	Impacts
	Mammals
	Reptiles
	Rights of Way
Linear Infrastructure	Biodiversity
	Biodiversity Mitigation
	Birds
	Collisions
	Electrocution
	Impacts
	Mammals
	Reptiles
	Rights of Way
Transmission Lines	Biodiversity
	Biodiversity Mitigation
	Birds
	Collisions
	Electrocution
	Impacts
	Mammals
	Reptiles
	Rights of Way

Each publication was analysed to determine relevance to each of the study questions by using a detailed search of the following keywords: “collision,” electrocution,” “linear infrastructure,” “rights of way (ROW)” and “transmission line,” while accounting for variances (synonyms) of each search term. Database searches were conducted between 5 December 2023 and 11 April 2024. Note that some publications from private organizations (i.e. consulting companies) were not accessible and therefore omitted.

**Figure 1.**
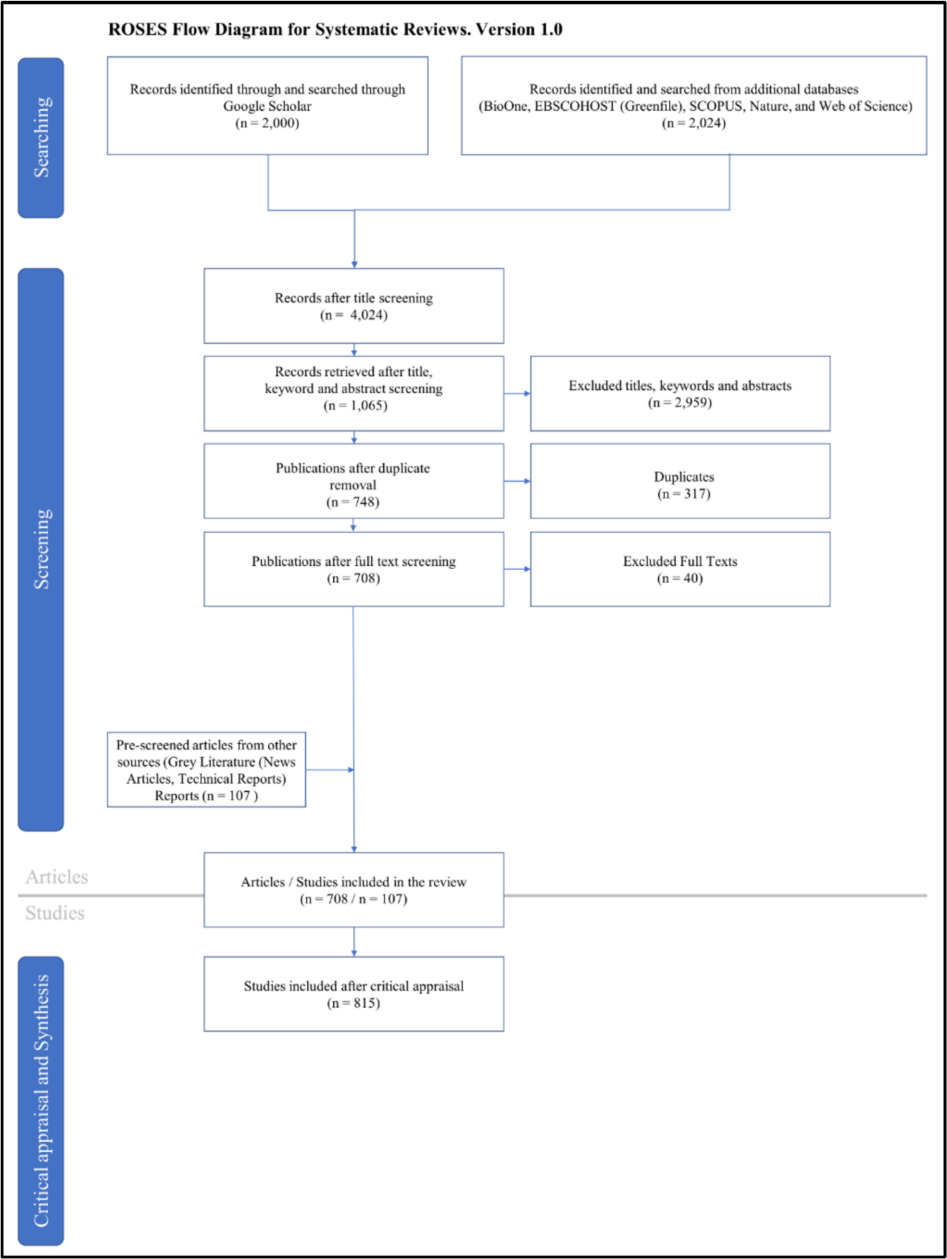
ROSES flow diagram outlining the screening of animal biodiversity and electrical infrastructure literature, resulting in the final list of studies that were retained in the review (Haddaway et al. 2015).

The Google Scholar search yielded 87,090 publications, which were examined based on their relevance to the search criteria at the title and abstract level, resulting in an initial 2,000 publications, which were then further refined at the document level resulting in 549 publications. A further 2,024 publications were then examined from the supplementary databases (BioOne, EBSCOHOST (Greenfile), SCOPUS, Nature, and Web of Science) and cross-referenced against the initial list from Google Schoolar, resulting in 474 publications found at the title and abstract level, which were then further refined to 159 publications after document-level assessment. Lastly, 107 publications were identified from grey literature sources, resulting in a final total of 815 publications chosen for critical appraisal (Fig. 1). Publications that conducted impact assessments focusing on single species were individually listed, but assessments directed towards more than one species were assigned into the category ‘Multiple Species Assessments.’ Large-scale assessments focusing on ecosystem and population responses, incorporating a wide variety of taxa, were assigned to the taxon category “Wildlife.”

To associate publications with the research objectives, each publication was categorised into one of the following topics: “impacts (positive and negative),” “impacts (positive),” “impacts (negative),” “mitigation measures,” “public engagement” and “research directives.” To determine knowledge gaps, publications from primary literature were further divided and assigned into impact categories (biotic alteration, collision/electrocution, construction management, disease transmission, habitat alteration, hunting, increased predation, invasive species, line alteration, noise, predictive modelling, spatial planning, strategic planning, tower alteration) (Table 2). Publications relating to ‘public engagement’ and ‘research directives’ were not assigned to further categories as they already had a specified focus. In addition, the following data were extracted from all papers: source type, taxon (class), IUCN status (single species assessments only), world region, and year of publication and retained in Microsoft Excel for statistical comparisons.

**Table 2:** Criteria used for classifying reviewed studies into impacts of electrical infrastructure on animal biodiversity

Impact	Category applied	Criteria/Definition
Biotic Alteration	Impacts (Positive), Impacts (Negative), Impacts (Positive and Negative)	Processes that causes change in metabolic processes e.g. Growth rate
Collision/Electrocution	Impacts (Negative)	Circumstances that result in direct impact with electrical infrastructure and passing of electrical current through an organism resulting in electrophysiological disruption or mortality
Construction Management	Mitigation Measures	Alterations made during construction phases to offset impacts
Disease Transmission	Impacts (Negative)	Any process that facilitates the spreads of disease
Habitat Alteration	Impacts (Positive), Impacts (Negative), Impacts (Positive and Negative)	Any circumstance that results in an increase or creation of habitat and associated resources required for organism survival. Includes use of electrical infrastructure by organisms through perching, nesting, roosting, and scavenging. Processes include: Corridor Creation, Edge Effects, Fragmentation, Habitat Loss
Hunting	Impacts (Negative)	Any circumstance that leads to direct injury/mortality of organisms through anthropogenic sources
Increased Predation	Impacts (Negative), Impacts (Positive and Negative)	Circumstances that leads to increased mortality of organisms through direct consumption
Invasive Species	Impacts (Negative)	Any circumstance/process that facilitates the spread of invasive species
Line Alteration	Management Strategies, Mitigation Measures	Any alteration to line structures to reduce collision/electrocution
Noise	Impacts (Negative)	Any alteration of organisms behaviour/movements during construction/operational phases
Predictive Modelling	Mitigation Measures	Processes used to predict future events/outcomes through analysis of selected input data
Spatial Planning	Management Strategies, Mitigation Measures	Methods and approaches used to offset negative impacts through spatial organisation
Strategic Planning	Management Strategies, Mitigation Measures, Research Directives	Methods and approaches used to offset negative impacts through changes to existing policy and guidelines
Tower Alteration	Management Strategies, Mitigation Measures	Any alteration to vertical structures to reduce collision/electrocution

## Results

**Fig. 2:**
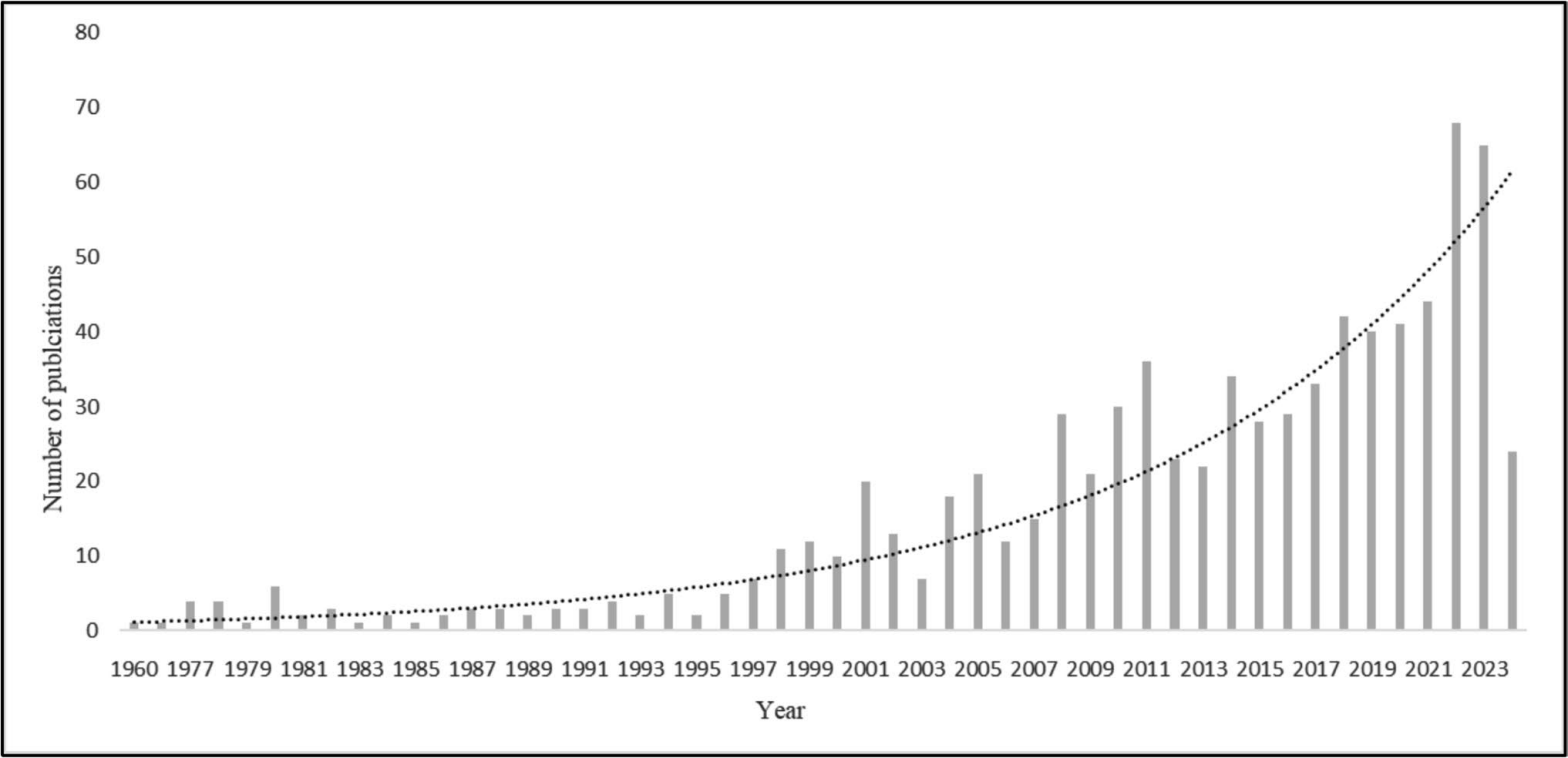
Temporal trend of animal biodiversity and electrical infrastructure studies from 1951 to 2024

**Fig. 3:**
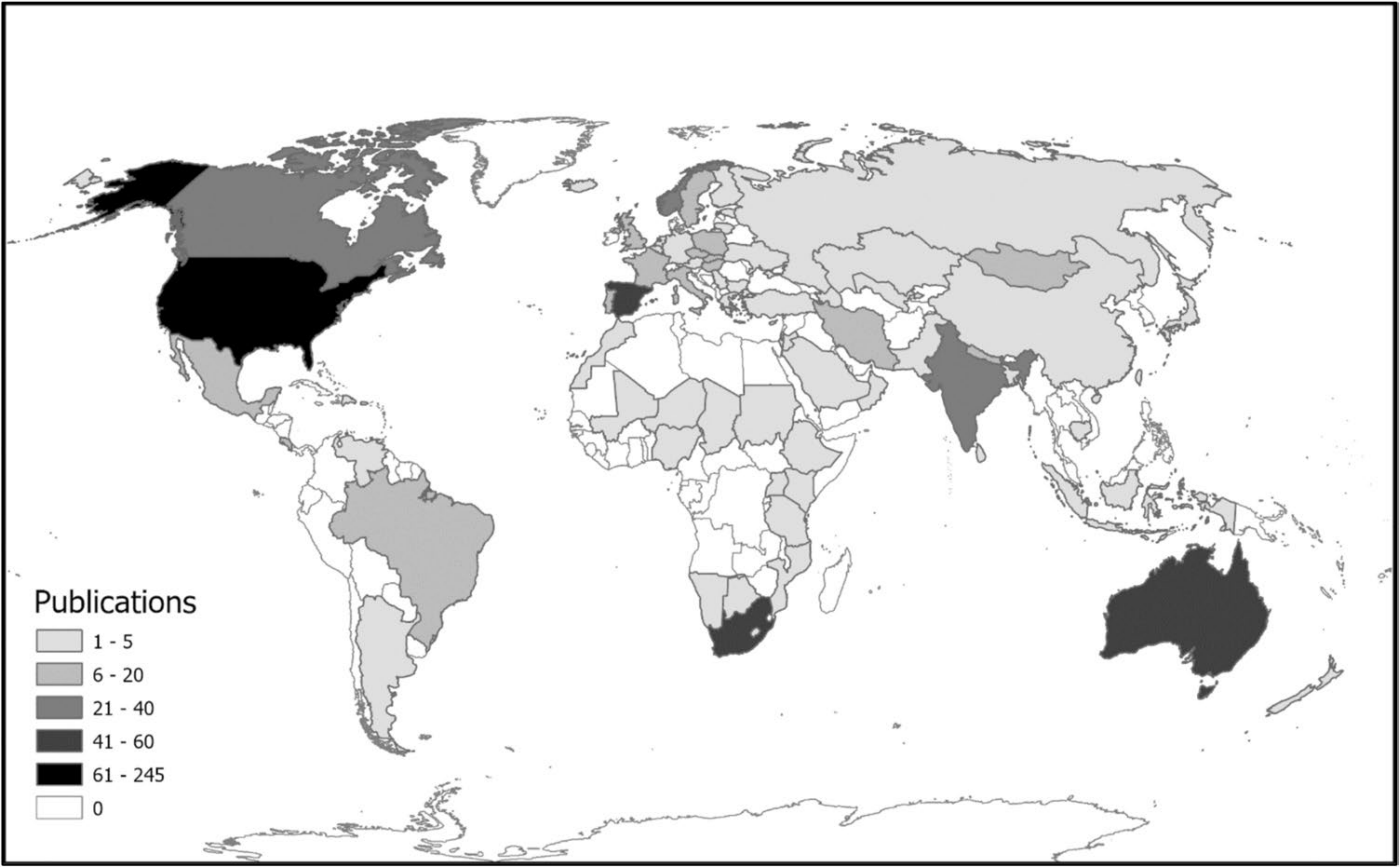
Spatial distribution of the 815 animal biodiversity and electrical infrastructure studies that met the criteria for use in this review. Shading indicates relative number of studies

Of the 815 publications, 87% (*n* = 709) were from peer-reviewed journals and 13% (*n* = 106) were from grey literature (Table 3). Publications on the topic increased exponentially over time from 1951 to the present (Fig. 2) and covered a wide range of animal groups (amphibians, birds, gastropods, insects, mammals, and reptiles). Studies were conducted across 71 countries and all continents except Antarctica, with the highest concentrations in North America and Europe along with three outliers — Australia, India, and South Africa, and the lowest from Africa, Asia, Oceania, and South America (Fig. 3). A small number of publications (*n* = 92) were found to conduct global assessments (*n* = 64) and assessments spanning multiple countries and continents (*n* = 28) incorporating major avian flyways (African-Eurasian, East Asian-Australasian, Eastern Mediterranean). Of these continental assessments, Europe (*n* = 19) and Africa (*n* = 14) were found to be covered the most.

**Table 3:** Percentage of literature (primary and grey) represented by each taxon (Class)

Taxon (Class)	Count	Percent
Amphibia	8	0.98
Aves	630	77.30
Gastropoda	2	0.25
Insecta	16	1.96
Mammalia	117	135
Reptilia	20	2.45

Literature that was found to assess multiple taxa were counted in each respective class total

Of the 815 publications identified, birds were found to be the most represented taxon, followed by mammals. Amphibians, gastropods, insects, and reptiles were represented but in relatively small proportions (Table 2). A large proportion of publications (*n* = 540) were found to conduct multiple species assessments accounting for 66% of the total literature assessed. Of these publications, the majority (75%) were found to assess birds (*n* = 406), generally focusing on specific taxonomic assessments of larger-bodied groups: bustards (Otidae), cranes (Gruidae), flamingos (Phoenicopteridae) grouse (Phasianidae), rails (Rallidae) and vultures (Cathartidae). Only a small proportion of publications (34%, *n* = 275) were found to conduct single-species assessments or multiple single-species assessments. Unsurprisingly, 81% (*n* = 224) of single species assessments were again focused on birds, with amphibians, gastropods, insects, mammals, and reptiles only comprising 19% (*n* = 51 of all single species assessments (Table 4). Again, a high proportion of single species assessments of birds (*n* = 199) focused on large-bodied or large-winged species: bustards and allies (Otidae), cranes (Gruidae), flamingos (Phoenicopteridae), geese (Anatidae), grouse (Phasianidae), ibises (Threskiornithidae), new world quail (Odontophoridae), pelicans (Pelecanidae), petrels and shearwaters (Procellariidae), storks (Ciconiidae), swans (Anatidae) and raptors (Accipitridae, Cathartidae*,* Falconidae*,* Strigidae*,* Tytonidae). A small proportion of the literature (*n* = 55) was found to assess broader wildlife (ecosystem and population) responses, accounting for 6.75% of all publications.

**Table 4:** Percentage of literature (primary and grey) represented by each taxon (Class) in regards to single species assessments

Taxon (Class)	Count	Percent
Amphibia	1	0.36
Aves	224	81.45
Gastropoda	1	0.36
Insecta	2	0.73
Mammalia	40	14.60
Reptilia	7	2.55

Literature that was found to conduct multiple individual species assessments were counted based on the number of individual assessments conducted

In terms of IUCN conservation status, we found a relatively even distribution between threatened (Critically Endangered (CR), Endangered (EN) and Vulnerable (VU)) and non-threatened (Near Threatened (NT), Least Concern (LC), Data Deficient (DD) and not evaluated (NE)) single species assessments, comprising 117 and 157 publications, respectively (Table 5). When solely looking at threatened species, birds comprised the majority of studies (*n* = 87) followed by mammals (*n* = 26) and then reptiles (*n* = 4). Similar circumstances were found regarding non-threatened species, with the majority of species again found to be predominantly birds (*n* = 136), followed by mammals (*n* = 14), reptiles (*n* = 3), insects (*n* = 2) amphibians (*n* = 1) and gastropods (*n* = 1). One insect, the red imported fire ant (*Solenopsis invicta*), could not be assigned an IUCN conservation status rating as it is an invasive species and excluded from IUCN evaluation, but was still retained for analysis and taxon-based counts.

**Table 5:** IUCN status and number of publications of species included in electrical infrastructure literature (primary and grey). References for this table can be found in Supplementary File S1

Taxon (Class)	Species	IUCN Status	Number of Publications	Citation
Amphibia	Blackbelly salamander (*Desmognathus quadramaculatus*)	LC	1	Cecala KK et al. (2014)
Aves	Bengal florican (*Houbaropsis bengalensis*)	CR	4	Jha RR et al. (2018) Mahood SP et al. (2018) Mahood SP et al. (2022) Sovannary S et al. (2022)
	Great Indian bustard (*Ardeotis nigriceps*)	CR	1	Kukreti I (2020)
	Newell’s shearwater (*Puffinus newelli*)	CR	7	Cooper BA, Day RH (1998) Day RH, Cooper BA (2022) Podolsky R et al. (1998) Raine AF et al (2017) Raine AF (2023) Travers M et al. (2021) Young LC et al. (2023)
	Lesser florican (*Sypheotides indicus*)	CR	1	Ram M et al. (2023)
	Ridgway’s hawk (*Buteo ridgwayi*)	CR	1	Dwyer JF et al. (2019)
	Chaco eagle (*Buteogallus coronatus*)	EN	2	Galmes MA et al. (2018) Sarasola JH et al. (2020)
	Great bustard (*Otis tarda*)	EN	12	Alonso JC et al. (2005) Hellmich J, Idaghdour Y (2002) Janss GF, Ferrer M (2000) Lane SJ et al. (2001) Lóránt M, Vadász C (2014) Lutkens R, Eder F (1977) Özgencil İK et al. (2022) Palacín C et al (2017) Palacin C et al. (2023) Raab R et al. (200) Raab R et al. (2011) Wang Y et al. (2022)
	Egyptian vulture (*Neophron percnopterus*)	EN	8	Angelov I et al. (2013) Arkumarev V (2014) Badia‐Boher JA et al. (2019) Donázar JA et al. (2002) Gangoso L, Palacios CJ (2002) McGrady MJ et al. (2024) Oppel S et al. (2021) Shobrak M (2020)
	Hawaiian petrel (*Pterodroma sandwichensis*)	EN	5	Cooper BA, Day RH (1998) Day RH, Cooper BA (2022) Raine AF et al (2017) Travers M et al. (2021) Young LC et al. (2023)
	Lear’s macaw (*Anodorhynchus leari*)	EN	1	Biasotto LD (2023)
	Ludwig’s bustard (*Neotis ludwigii*)	EN	3	Evans SW (2023) Jenkins AR et al. (2011) Shaw JM (2013)
	Martial eagle (*Polemaetus bellicosus*)	EN	1	Amar A, Cleote D (2018)
	Northern bald ibis (*Geronticus eremita*)	EN	1	Fritz J et al (2017)
	Saker falcon (*Falco cherrug*)	EN	2	Dixon A (2020) Puzović S (2008)
	Steppe eagle (*Aquila nipalensis*)	EN	1	Shobrak M (2022)
	Whooping crane (*Grus americana*)	EN	6	Ellis KS (2022) Folk MJ et al. (2008) Folk MJ (2013) Ivey G (2014) Miller JL et al. (2010) Stehn TV, T Wassenich (2008)
	Blue crane (*Grus paradisea*)	VU	2	Kotoane M (2004) Shaw JM et al. (2010)
	Cape vulture (*Gyps coprotheres*)	VU	5	Boshoff AF et al. (2011) Bromfield M (2019) Ledger JA, Annegarn HJ (1981) Martens FR (2018) Phipps WL (2013)
	Houbara bustard (*Chlamydotis undulata*)	VU	3	Abril-Colon I et al. (2022) Alonso JC (2024) Dolman P M et al. (2021)
	Lesser prairie-chicken (*Tympanuchus pallidicinctus*)	VU	10	Hagen CA et al. (2011) Hagen CA et al. (2007) Ott JP, et al. (2021) Patten MA et al. (2021) Peterson JM et al. (2020) Plumb RT et al. (2019) Pruett CL et al. (2009) Robinson SG (2018) Wolfe DH et al. (2007) Wolfe DH et al. (2016)
	Macqueen’s bustard (*Chlamydotis macqueenii*)	VU	2	Burnside R et al. (2015) Burnside RJ et al. (2018)
	Sarus crane (*Antigone antigone*)	VU	2	Sharma HP et al. (2024) Sundar KSG, Choudhury BC (2005)
	Spanish imperial eagle (*Aquila adalberti*)	VU	7	Bisson IA et al. (2002) Ferrer M, Hiraldo F (1991) Ferrer M, Hiraldo F (1992) Ferrer M (2001) The Iberian Imperial Eagle, Lynx Nature Books González LM et al. (2007) Janss GF, Ferrer M (2001) Lopez-Peinado A et al. (2023)
	Bearded vulture (*Gypaetus barbatus*)	NT	4	Kruger SC, Amar A (2017) Krüger SC, Amar A (2021) Margalida A et al. (2008) Yousefi M et al. (2023)
	Cerulean warbler (*Setophaga cerulea*)	NT	1	Bohall-Wood P et al. (2006)
	Dalmatian pelican (*Pelecanus crispus*)	NT	1	Crivelli AJ et al. (1988)
	Golden-winged warbler (*Vermivora chrysoptera*)	NT	1	Klaus NA, Buehler DA (2001)
	Greater sage-grouse (*Centrocercus urophasianus*)	NT	13	Baxter JJ et al (2017) Braun CE (1998) Dwyer JF, Doloughan KW (2014) Fedy BC et al. (2015) Gibson D et al. (2018) Hansen EP et al. (2016) Kirol CP (2015) Kohl MT (2019) Messmer TA (2013) Naugle DE (2006) Picardi S (2020) Schroeder MA (2010) Walker BL (2008)
	Little bustard (*Tetrax tetrax*)	NT	10	Manosa S (2023) Marcelino J et al. (2018) Marques AT et al. (2020) Morales MB et al. (2005) Morales, MB, Bretagnolle V (2022) Santangeli A et al. (2023) Silva JP et al. (2010) Silva JP et al. (2014) Silva JP et al. (2022) Silva JP et al. (2023)
	Northern bobwhite (*Colinus virginianus*)	NT	1	Dunkin SW et al. (2009)
	Red kite (*Milvus milvus*)	NT	1	Tavecchia G et al. (2012)
	American kestrel (*Falco sparverius*)	LC	3	Fernie KJ, Bird DM (1999) Fernie KJ et al. (2000) Morrow L, Morrow J (2018)
	Bald eagle (*Haliaeetus leucocephalus*)	LC	6	Brand CJ (2013) Dell DA, Zwank PJ (1986) Harness RE (2004) Mojica EK (2009) Riley L (2014) Watts BD et al. (2015)
	Barn owl (*Tyto alba*)	LC	1	Álvarez-Castañeda ST et al. (2004)
	Black-necked stork (*Ephippiorhynchus asiaticus*)	LC	2	Clancy GP (2010) Clancy GP (2011)
	Black stork (*Ciconia nigra*)	LC	1	Smeraldo S (2020)
	Bonelli’s eagle (*Aquila fasciata*)	LC	9	Chevallier C et al. (2015) Dias A et al (2017) Hernandez-Matias A et al. (2015) Lopez-Peinado A et al. (2023) Manosa S, Real J (2001) Marques AT et al. (2022) Real J et al. (2001) Rollan A et al. (2010) Soutullo A et al. (2008)
	Brolga (*Antigone rubicunda*)	LC	1	Veltheim I et al. (2019)
	Common buzzard (*Buteo buteo*)	LC	1	Kalpakis S et al. (2009)
	Common crane (*Grus grus*)	LC	2	Fanke J et al. (2011)
	Common nighthawk (*Chordeiles minor*)	LC	1	Hausleitner D, Wallace J (2012)
	Common raven (*Corvus corax*)	LC	1	Shurtliff QR, Whiting JC (2021)
	Common tern (*Sterna hirundo*)	LC	1	Henderson IG et al. (1996)
	Demoiselle crane (*Grus virgo*)	LC	1	Mali S et al. (2023)
	Eastern grass owl (*Tyto longimembris*)	LC	1	Samson A, Kumar PS (2015)
	Eurasian eagle owl (*Bubo bubo*)	LC	8	Husby M, Pearson M (2022) Martinez JA et al. (2006) Mikkola H, Tornberg R (2014) Nygård T et al. (2023) Pérez-García JM et al. (2016) Rubolini D et al. (2001) Schaub M et al. (2010) Sergio F et al. (2004)
	Eurasian griffon vulture (*Gyps fulvus*)	LC	1	Vulture Conservation Foundation (2022)
	Eurasian kestrel (*Falco tinnunculus*)	LC	3	Dell'Omo G et al. (2009) Kolnegari M et al. (2020) Sos-Koroknai V et al. (2020)
	European roller (*Coracias garrulus*)	LC	1	Malovichko LV (2023)
	Ferruginous hawk (*Buteo regalis*)	LC	2	Gilmer DS, Stewart R (1983) Parayko NW (2021)
	Golden eagle (*Aquila chrysaetos*)	LC	7	Allison TD et al (2017) Brand CJ (2013) Dwyer JF et al (2017) Kochert MN et al. (2019) Mojica EK et al. (2018) Viner TC et al. (2022) Wiens JD (2017)
	Grasshopper sparrow (*Ammodramus savannarum*)	LC	1	Allen MC et al. (2022)
	Great tit (*Parus major*)	LC	1	Tomas G et al. (2012)
	Greater flamingo (*Phoenicopterus roseus*)	LC	1	Kurhade S (2017)
	Greater white-fronted goose (*Anser albifrons*)	LC	1	Shimada T (2001)
	Harris hawk (*Parabuteo unicinctus*)	LC	1	Dwyer JF (2006)
	Henslow’s sparrow (*Centronyx henslowii*)	LC	2	Dwire AW (2021) Hunter EA et al. (2022)
	Hooded robin (*Melanodryas cucullata*)	LC	1	Fitrp LL, Ford HA (1997)
	Long-legged buzzard (*Buteo rufinus*)	LC	1	Kalpakis S et al. (2009)
	Meadow bunting (*Emberiza cidoides*)	LC	1	Wen-hong D et al. (2003)
	Monk parakeet (*Myiopsitta monachus*)	LC	1	Newman JR et al. (2008)
	New Zealand falcon (*Falco novaeseelandiae*)	LC	1	Fox NC, Wynn C (2010)
	Northern pintail (*Anas acuta*)	LC	1	Guyn KL, Clark RG (2000)
	Oriental dollarbird (*Eurystomus orientalis*)	LC	1	Wiles GJ et al. (2020)
	Osprey (*Pandion haliaetus*)	LC	1	Murphy N K et al. (2024)
	Ovenbird (*Seiurus aurocapilla*)	LC	1	Bayne EM, Hobson KA (2001)
	Peregrine falcon (*Falco peregrinus*)	LC	1	Gahbauer, MA (2015)
	Pied crow (*Corvus albus*)	LC	2	Cunningham SJ (2016) Senoge ND, Downs CT (2003)
	Pink-footed goose (*Anser brachyrhynchus*)	LC	1	Wood KA (2020)
	Prairie warbler (*Setophaga discolor*)	LC	1	Akresh ME et al. (2015)
	Red-backed shrike (*Lanius collurio*)	LC	1	Bakx TR et al. (2020)
	Sandhill crane (*Antigone canadensis*)	LC	9	Dwyer JF et al. (2019) Fannin TE (1992) Hays QR et al. (2021) Morkill AE (1990) Morkill AE, Anderson SH (1991) Murphy RK et al. (2016) Murphy RK et al. (2016) Ward JP, SH Anderson (1992) Windingstad RM (1988)
	Scissor-tailed flycatcher (*Tyrannus forficatus*)	LC	1	Roeder DV et al. (2022)
	Scrub euphonia (*Euphonia affinis*)	LC	1	Brush T (2009)
	Verreaux’s eagle (*Aquila verreauxii*)	LC	1	Symes CT, Kruger TL (2012)
	Wedge-tailed eagle (*Aquila audax*)	LC	1	Bekessy SA et al. (2009)
	White-bellied sea eagle (*Icthyophaga leucogaster*)	LC	1	Byju, H et al. (2023)
	White-tailed eagle (*Haliaeetus albicilla*)	LC	1	Cole SG, Dahl EL (2013)
	Williamson’s sapsucker (*Sphyrapicus thyroideus*)	LC	1	St-Amand J et al. (2021)
	Willow ptarmigan (*Lagopus lagopus*)	LC	1	Smith A, Willebrand T (1999)
	Wood thrush (*Hylocichla mustelina*)	LC	1	Kaiser SA, Lindell, CA (2007)
	Yellow-breasted chat (*Icteria virens*)	LC	1	Lehnen SE, Rodewald AD (2013)
	Yellowhammer (*Emberiza citrinella*)	LC	1	Bakx TR et al. (2020)
	White stork (*Ciconia ciconia*)	LC	9	Burdett EM et al. (2022) Garrido JR, Fernández-Cruz M.(2003) Kaługa I et al. (2011) Marcelino J et al. (2021) Moreira F et al (2017) Moreira F et al. (2018) Tobolka M (2014) Vaitkuvienė D, Dagys M (2014)
Gastropoda	Lemon snail (*Cepaea nemoralis*)	LC	1	Ossenkopp KP et al. (1990)
Insecta	Red imported fire ant (*Solenopsis invicta*)	NE	2	Stiles JH, Jones RH (1998) Todd B D et al. (2008)
Mammalia	Asian elephant (*Elephas maximus*)	EN	2	Palei NC et al. (2014) Palei NC et al. (2015)
	Crab-eating macaque (*Macaca fascicularis*)	EN	1	Loudon JE et al. (2024)
	Golden lion tamarin (*Leontopithecus rosalia*)	EN	1	Lucas PDS et al. (2019)
	Mahogany glider (*Petaurus gracillis*)	EN	2	Asari YJ et al. (2010) Jackson SM et al. (2020)
	Brown howler (*Alouatta guariba*)	VU	2	Corrêa FM et al. (2018) Monticelli C et al. (2022)
	Grey-headed flying fox (*Pteropus poliocephalus*)	VU	2	Mo M et al. (2023) Tidemann CR, Nelson JE (2011)
	Koala (*Phascolarctos cinereus*)	VU	3	De Oliveira et al. (2014) De Oliveira SM et al. (2014) Koefoed L (2024)
	Mantled howler (*Alouatta palliata*)	VU	1	Rojas IA, Gregory T (2022)
	Reindeer (*Rangifer tarandus*)	VU	12	Colman JE et al. (2015) Colman JE et al (2017) Dawe DA et al. (2022) Eftestøl S et al. (2016) Flydal K et al. (2009) Nellemann C et al. (2001) Reimers E et al. (2007) Risvoll C et al. (2022) Tyler NJ et al. (2016) Vistnes I, Nellemann C (2001) Vistnes I et al. (2004)
	Broad-toothed mouse (*Mastacomys fuscus*)	NT	1	Clarke DJ, White JG (2008)
	Yellow-bellied glider (*Petaurus australis*)	NT	1	Kambouris PJ et al. (2013)
	Brown bear (*Ursus actos*)	LC	1	Desmecht B (2017)
	Elk (*Cervus canadensis*)	LC	1	Canfield JE (1984)
	Grey wolf (*Canis lupus*)	LC	1	Paquet PC, Callahan C (1996)
	Indian flying fox (*Pteropus medius*)	LC	1	Tella JL et al. (2020)
	Moose (*Alces alces*)	LC	4	Bartzke GS (2014) Bartzke GS et al. (2015) Neumann W et al. (2013) Ricard JG, Doucet GJ (1999)
	Ringtail possum (*Pseudocheirus peregrinus*)	LC	2	Blasdell KR (2022) Wilson RF et al. (2007)
	White-tailed deer (*Odocoileus virginianus*)	LC	2	Doucet GJ, Thompson ER (2008) Rieucau G et al. (2007)
Reptilia	Agassiz’s desert tortoise (*Gopherus agassizii*)	CR	1	Berry KH et al. (2020)
	Spotted turtle (*Clemmys guttata*)	EN	2	Litzgus JD, Mousseau TA (2004) Litzgus JD, Mousseau TA (2006)
	Gopher tortoise (*Gopherus polyphemus*)	VU	1	Jodice PGR et al. (2006)
	Eastern hognose snake (*Heterodon platirhinos*)	LC	2	Akresh ME et al (2017) Plummer MV (2002)
	Stokes’s skink (*Egernia stokesii*)	LC	1	Bradley HS et al. (2023)

When assessing the impacts of electrical infrastructure on animal biodiversity, we found that the majority of publications (*n* = 489) reported that powerlines and electrical infrastructure negatively impacted animal biodiversity accounting for 60% of all publications. Of these, collision/electrocution (*n* = 281) and habitat alteration (*n* = 156) were determined to be the primary factors impacting animal biodiversity, while only two publications documented the impacts of noise, focusing specifically on reindeer (*Rangifer tarandus*). This was further supported by a large number of publications (*n* = 318) focusing on mitigation, predominantly conducting line alterations (*n* = 163) and tower alterations (n 96). Only a small proportion of publications addressing mitigation were found to address impacts during the construction phase (*n* = 8) (Table 6). However, while our study found a myriad of mitigation measures, we found no publications addressing the mitigation of electromagnetic fields (EMF) despite several publications documented negative impacts (*n* = 18). Further to this, only a handful of publications (*n* = 6) were identified to assess the impacts and mitigation of ultraviolet radiation (UV).

**Table 6:** Percentage of mitigation literature (primary and grey) in regards to publication focus

Publication Focus	Count	Percent
Construction Management	8	2.51
Habitat Alteration	45	13.79
Line Alteration	163	51.41
Predictive Modelling	43	13.48
Spatial Planning	87	27.59
Strategic Planning	61	19.12
Tower Alteration	96	30.41

Literature that was found to incorporate multiple topics was counted in each respective focus total

In comparison, publications that reported the positive effects of electrical infrastructure on animal biodiversity were found to be much less (*n* = 166) accounting for only 20% of the total literature. Of these publications, we found that habitat alteration (*n* = 161) was the most prominent factor impacting animal biodiversity followed by biotic alteration (*n* = 8).

We found that only a handful of publications (*n* = 63) found that electrical infrastructure provided both positive and negative impacts on animal biodiversity, documenting the effects of habitat alteration (*n* = 60) increased predation (*n* = 14) and biotic alteration (*n* = 5). All publications that reported collision/electrocution-based impacts were also found to document habitat alteration as a positive impact (*n* = 22).

## Discussion

In this review, we systematically assessed the literature relating to electrical infrastructure and animal biodiversity. Our results indicate that knowledge around electrical infrastructure and animal biodiversity has been growing exponentially over the past 25 years, most likely due to the high demand and transition towards renewable energy sources, but there remain significant knowledge gaps. This was confirmed by the fact that the most recent empirical studies are from the United States and some parts of Europe, along with a handful of economically developed countries (Australia, Canada, Norway, and South Africa). Unsurprisingly, we found that Europe was the most represented in terms of continental and global assessments, specifically Western Europe, as the majority of countries already have well-developed electrical infrastructure networks to coincide with the energy demand from high population densities. Surprisingly, we found Africa to be one of the most represented continents given that there are large expanses of areas without access to electrical infrastructure networks. However, given that the Palearctic-African flyways collectively support the world’s largest migration system, we suggest this has drawn a significant proportion of research output (BirdLife International 2010). However, the number of publications found to conduct continental and global assessments overall was found to be low, indicating our knowledge regarding flyways and migratory species is still poor, most likely due to large logistical requirements and cooperative investment needed from multiple countries. Similarly, we found that only a very small proportion of publications conducted assessments across a wide range of animal species in an affected area, indicating that our knowledge towards community/ecosystem-based responses is very limited, most likely due, again, to the large amounts of resources required to attain long-term responses of species, populations, and environmental processes.

When considering the impacts of electrical infrastructure, we found that the majority of impacts towards animal biodiversity were reported to be negative. This was primarily due to barrier (collision) effects and resultant electrocution from use as a (perching) resource. This indicates that while mitigation measures are widely available, implementation is perhaps not as widespread due to current geographical biases or their general effectiveness to offset the current negative impact. This was further highlighted by our knowledge of EMF, noise and UV mitigation still being largely unstudied.

On the scale of populations, we found that collision and electrocution-based mortality can have significant effects on specific species such as black grouse (*Lyurus tetrix*), capercaillie (*Tetrao urogallus*) and willow ptarmigan (*Lagopus lagopus*) in Norway, with national loss estimates of 26,000, 20,000 and 50,000 individuals respectively, which were found to be significantly higher than hunting-based mortality estimates (Bevanger 1995; Bevanger and Brøseth 2004). We also found that electrocution/collision was the main driving factor behind significant population declines of European raptors including: Bonelli’s eagle (*Aquila fasciata*), Eurasian eagle owl (*Bubo bubo*) and the Spanish imperial eagle (*Aquila aldaberti*) along with Ludwig’s bustard (*Neotis lugwigii*) in South Africa (Sergio et al. 2004; Mariano González et al. 2007; Schaub et al. 2010; Jenkins et al. 2011; Hernández-Matías et al. 2015). Moreover, we found electrical infrastructure, specifically power lines, posed a significant impact to important migratory (Central Asian Flyway (CAF)) routes, contributing to population declines of 14 threatened species, including the critically endangered great Indian bustard (*Ardeotis nigriceps*) estimated to have a mortality rate of 16% per year due to electrical infrastructure (Uddin et al. 2021).

On the other hand, we found electrical infrastructure has the potential to provide significant benefits to certain species, supporting population increases of pied crows (*Corvus albus*) and white stork through provisioning of additional perching spots and nesting areas (Moreira et al. 2017). However, while we found electrical infrastructure to stimulate population increase of generalist predators such as pied crows, this often expedited local population declines of angulate tortoise (*Chersina angulate*) in South Africa through hyper-predation, similar to the interactions found between the common raven (*Corvus corvax*) and Agassiz’s desert tortoise (*Gopherus agassizii*) in the United States, indicating that electrical infrastructure facilitates a “winner and loser effect” through reduction of prey refugia efficacy (Lovich and Ennen 2013; DeMars and Boutin 2017; Cunningham et al. 2015; Berry et al. 2020).

We detected a clear taxonomic bias towards birds and, to a lesser extent, mammals, when assessing single species impacted by electrical infrastructure. This was to some extent expected as the literature suggests that larger-bodied taxa have a higher predisposition to collisions and electrocutions heightened by those species that traverse and interact with electrical infrastructure through various behaviours (hunting, perching, and roosting) (Birdlife International 2017). This suggestion was further supported by a large proportion of mitigation literature focusing on reducing barrier effects through line and tower alterations. Another possible explanation for our observed taxonomic bias is that large-bodied taxa are easier to detect, as opposed to smaller-bodied taxa, allowing for more reliable and scalable population assessments. Given this detection probability, it was not surprising that we found only a small number of studies focused on amphibians, gastropods, insects, and reptiles—those species groups that have generally reduced mobility and increased sensitivity to environmental alteration such as how electrical infrastructure can alter Rights of Way (RoW) through habitat alteration and edge-based effects (Ferreira et al. 2016).

Given that electrical infrastructure networks often cover thousands of kilometres in length, it is often not pragmatic to conduct full assessments of mortality, with only a small proportion of studies conducting rigorous systematic assessments. This has resulted in a large amount of data being collected opportunistically from isolated incidents, often based on convenience (ability to access the infrastructure) or from assessments of small proportions of linear infrastructure networks. This bias has led to scaling issues regarding national and population mortality estimates of threatened species such as the Cape Vulture (*Gyps coprotheres*) (Uddin et al.^2021^). Furthermore, the mortality rate of collision and electrocution in the United States is estimated at between 12 and 64 million birds, confirming that assessments can severely over- or under-estimate mortality statistics (Rioux et al. 2013; Loss et al. 2014a, b).

Given the limited accuracy of current assessments, comparisons of electrical infrastructure with other sources of anthropogenic mortality can still be made, allowing scientists and practitioners to prioritise mitigation measures given limited resource availability. When comparing mortality across anthropogenic sources, we found that mortality via electrical infrastructure ranks relatively low in comparison to predation by domestic/feral cats (identified to be the most prominent driver of decline with an estimated 1.4–4 billion birds lost annually in the United States). This was followed by building and automobile collisions with annual mortality estimates of 100,000,000–1000,000,000 and 200,000,000–340,000,000, respectively. In terms of comparisons with other energy infrastructure sources, power lines pose a significantly greater impact on animal biodiversity than monopole wind turbines (annual U.S. mortality estimates of 140,000–328,000) and terrestrial oil and gas development (annual U.S. mortality estimates of 9,900–72,000) (Klem Jr. 2009; Loss et al. 2013; Loss et al. 2014a, b; Loss 2016; Basilio et al. 2020). More concerningly, our review supports the findings by Loss (2016) of expected proportionate increases in collision-based mortality to mirror human population growth and energy demand, while continuing to encroach into areas previously unaffected by anthropogenic activity that support Key Biodiversity Areas (KBAs) and Key Protected Areas (KPA’s). Simkins et al. (2023) indicate that powerlines already impact 36.7% of global KBAs and are predicted to increase as transport, energy, extractives, urban areas and water reservoirs increase in the future. Given that a large proportion of future renewable energy developments are predicted to occur predominantly in Indonesia and South America, which support significantly high biodiversity, coupled with a small proportion of our findings from these areas (which could be due to lack of searches in publications in Indonesian and Spanish), we suggest these areas will rapidly become focal areas for future research (Myers et al. 2000; Rehbein et al. 2020).

### Future recommendations

With the majority of current literature indicating the negative impacts of electrical infrastructure, there is considerable potential for improvement with even modest modifications shown to greatly improve biodiversity (Ferrer et al. 2020). However, given that current literature is confined primarily to developed countries we call for more comprehensive global assessments. As practitioners often have to work within limited resources in order to maximise conservation efficiency, we suggest focusing on tropical regions indicative of high biodiversity and areas predicted to undergo rapid renewable energy expansion (Wilson et al. 2006). In addition, we call for more assessments of migratory species that traverse multiple countries and which are subject to a wider variety of electrical infrastructure amongst additive effects from factors such as climate change and habitat loss and larger-bodied species predisposed to collision and electrocution-based mortality (Runge et al. 2015; Zurell et al. 2018).

As we found limited research output regarding less mobile taxonomic groups (amphibians, gastropods, insects, and reptiles) sensitive to environmental alteration, it is only natural that we call for increased representation given current taxonomic biases to further delineate the magnitude of impacts on communities and ecosystem processes (Rosenthal et al. 2017). Furthermore, given that we also found a relatively even distribution of non-threatened and threatened species studies, we encourage future assessments not to directly focus on threatened species and continue to make assessments when logistically possible as this would prevent studies of common species that could be more representative of ecosystem function (Lyons et al. 2005).

While we only found a small proportion of publications documenting that electrical infrastructure, specifically powerlines, can create a ‘winner and loser effect’ resulting in increases of species and declines of others, there needs to be significant expansion into additional taxonomic groups to determine whether these processes are species-specific or community wide. Moreover, there is a need to determine the causative factors behind these processes, such as resource availability, increased competition, or facilitating movements of invasive species by (Filgueiras et al. 2021).

At the project scale, our results further support the findings by Biasotto and Kindel (2018), indicating that the majority of Environmental Impact Assessments (EIAs) and subsequent mitigation measures are conducted during the operational phase, omitting pre-construction phases and the cumulative effects posed on surrounding biodiversity. We therefore call for more aggressive EIA scoping phases and development of a standardised systematic process to allow assessment of immediate and longer-term impacts across all phases of operation on populations given that knowledge is still poor in this area (Bigard et al. 2017). Future projects should also be encouraged to engage with all stakeholders before development and work alongside citizen science programs, increasing public cooperation when possible, granting access to point data records, which can be used to determine sensitive areas through GIS analyses, identifying hotspots of threatened species and areas, ensuring safe spatial planning (Messmer et al. 2013; Paquet et al. 2022).

Finally, with a large proportion of operational phase assessments looking at the impacts and mitigation around collision and electrocution, future research would greatly benefit by expanding into EMF, noise, and UV radiation to provide a more comprehensive understanding of electrical infrastructure. In terms of management of electrical infrastructure and associated ROW, introducing varying successional vegetation schemes should be incorporated, when possible, to create habitat heterogeneity and provide maximum potential for sustaining biodiversity (Clarke et al. 2006).

## Conclusion

Our review reveals that electrical infrastructure, specifically powerlines can pose significant negative effects to animal biodiversity, primarily through collision and electrocution, but also provide positive effects, such as habitat provisioning. However, there remain significant knowledge gaps and taxonomic biases that need to be addressed to understand the full extent of impacts and wider effects on populations and ecosystem processes. In order to address these caveats, scientists and practitioners require adequate resources to develop and conduct standardised systematic assessments of electrical infrastructure, working alongside developers and engineers throughout planning, installation, and operational phases. The resultant outcomes can then subsequently be used by corporations, governmental organisations, and policy makers to establish best practice and form global mitigation guidelines to avoid additional species losses, helping prioritise the losses posed by highly degradative processes such as climate change, habitat alteration, habitat destruction and habitat loss. Finally, given that the majority of impacts from powerlines are similar to those posed by anthropogenic energy production sources (collision with wind turbines, environmental damage from coal-fired power stations and nuclear power stations), the results in this review can be adapted to inform critical assessments of other sources of anthropogenic mortality, particularly those linear in nature.

## Supplementary Information

Below is the link to the electronic supplementary material.

Supplementary file1 (DOCX 81 KB)

## Data Availability

Available from the corresponding author on reasonable request.
