# Correction: Positive and negative impacts of electrical infrastructure on animal biodiversity: A systematic review

**DOI:** 10.1007/s00442-025-05804-2

**Published:** 2025-10-13

**Authors:** Adam J. Bennett, David M. Watson, Maggie J. Watson

**Affiliations:** 1 Charles Sturt University, 386 Elizabeth Mitchell Drive, Thurgoona, NSW 2640 Australia; 2 Bird Group, The Natural History Museum, Tring, Hertfordshire HP23 6AP UK

**Correction: Oecologia (2025) 207:142** 10.1007/s00442-025-05780-7

In Table 6 of this article, the data in the column headed Count were mistakenly listed as 878 instead of 87. The old incorrect and the corrected version of Table 6 given below.

The original article has been corrected.

Incorrect version:

**Table:** **Table 6** Percentage of mitigation literature (primary and grey) in regards to publication focus

Publication Focus	Count	Percent
Construction Management	8	2.51
Habitat Alteration	45	13.79
Line Alteration	163	51.41
Predictive Modelling	43	13.48
Spatial Planning	878	27.59
Strategic Planning	61	19.12
Tower Alteration	96	30.41

Literature that was found to incorporate multiple topics was counted in each respective focus total

Corrected version:

**Table:** **Table 6** Percentage of mitigation literature (primary and grey) in regards to publication focus

Publication Focus	Count	Percent
Construction Management	8	2.51
Habitat Alteration	45	13.79
Line Alteration	163	51.41
Predictive Modelling	43	13.48
Spatial Planning	87	27.59
Strategic Planning	61	19.12
Tower Alteration	96	30.41

Literature that was found to incorporate multiple topics was counted in each respective focus total

