# Supplementary material for: Positive and negative impacts of electrical infrastructure on animal biodiversity: A systematic review
Source: Oecologia. 2025 Aug 13;207(9):142. doi: 10.1007/s00442-025-05780-7 (PMC12350552; doi:10.1007/s00442-025-05780-7)
Supplement: Supplementary file 1 — Supplementary file1 (DOCX 81 KB) [file 442_2025_5780_MOESM1_ESM.docx]

Full Reference List (Primary and Grey) – Supplementary File 1

Primary

Abril-Colon I, Alonso JC, Palacin C, Alvarez-Martinez JM, Ucero A (2022) Short-distance nocturnal migration in an island endemic bustard. Ibis, 164(4), 1145-1159.

Akresh ME, King DI, Timm BC, Brooks RT (2017) Fuels management and habitat restoration activities benefit Eastern Hognose Snakes (*Heterodon platirhinos*) in a disturbance-dependent ecosystem. Journal of Herpetology, 51(4), 468-476.

Akresh ME, King D, Brooks RT (2015) Demographic response of a shrubland bird to habitat creation, succession, and disturbance in a dynamic landscape. Forest Ecology and Management, 336, 72-80.

Allen MC, Almendinger T, Barreca CT, Lockwood JL (2022) A lidar-based openness index to aid conservation planning for grassland wildlife. Avian Conservation and Ecology, 17(1).

Allison TD, Cochrane JF, Lonsdorf E, Sanders-Reed C (2017) A review of options for mitigating take of Golden Eagles at wind energy facilities. Journal of Raptor Research, 51(3), 319-333.

Alonso JC, Abril-Colon I, Ucero A, Palacin C (2024) Anthropogenic mortality threatens the survival of Canarian houbara bustards. Scientific Reports 14(1), 2056.

Alonso JC, Martin CA, Palacin C, Martin B, Magana M (2005) The Great Bustard Otis tarda in Andalusia, southern Spain: Status, distribution and trends. Ardeola, 52(1), 67-78.

Álvarez-Castañeda ST, Cárdenas N, Méndez, L (2004) Analysis of mammal remains from owl pellets (*Tyto alba*), in a suburban area in Baja California. Journal of arid environments, 59(1), 59-69.

Amar A, Cleote D (2018) Quantifying the decline of the Martial Eagle *Polemaetus bellicosus* in South Africa. Bird Conservation International, 28(3), 363-374.

Andriushchenko YA, Popenko VM (2012) Birds and Power Lines in Steppe Crimea: Positive and Negative Impacts, Ukraine. Raptors Conservation, (24).

Angelov I, Hashim I, Oppel S (2013). Persistent electrocution mortality of Egyptian Vultures *Neophron percnopterus* over 28 years in East Africa. Bird Conservation International, 23(1), 1-6.

Arkumarev V, Dobrev V, Abebe YD, Popgeorgiev G, Nikolov SC (2014) Congregations of wintering Egyptian Vultures *Neophron percnopterus* in Afar, Ethiopia: present status and implications for conservation. Ostrich, 85(2), 139-145.

Asari Y, Johnson CN, Parsons M, Larson J (2010) Gap-crossing in fragmented habitats by mahogany gliders (*Petaurus gracilis*). Do they cross roads and powerline corridors? Australian Mammalogy, 32(1), 10-15.

Badia‐Boher JA, Sanz‐Aguilar A, De la Riva M, Gangoso L, Van Overveld T, García‐Alfonso M, Luzardo, OP, Suarez‐Pérez A, Donázar JA (2019) Evaluating European LIFE conservation projects: Improvements in survival of an endangered vulture. Journal of Applied Ecology, 56(5), 1210-1219.

Baena MF (2005) The Iberian Imperial Eagle. Ecosystems, 14(3).

Bakx TR, Lindström Å, Ram D, Pettersson LB, Smith HG, van Loon EE, Caplat P (2020) Farmland birds occupying forest clear-cuts respond to both local and landscape features. Forest Ecology and Management, 478, 118519.

Bartzke GS (2014) Effects of power lines on moose (*Alces alces*) habitat selection, movements and feeding activity. Norwegian University of Science and Technology. Faculty of Natural Sciences and Technology. Department of Biology.

Bartzke GS, May R, Solberg EJ, Rolandsen CM, Røskaf E (2015) Differential barrier and corridor effects of power lines, roads and rivers on moose (*Alces alces*) movements. Ecosphere, 6(4), 1-17.

Baxter JJ, Baxter RJ, Dahlgren DK, Larsen RT (2017) Resource selection by Greater Sage-Grouse reveals preference for mechanically-altered habitats. Rangeland Ecology & Management, 70(4), pp.493-503.

Bayne EM, Hobson KA (2001) Movement Patterns of adult male Ovenbirds during the post fledging period in fragmented and forested boreal landscapes. The Condor, 103(2), 343-351.

Bekessy SA, Wintle BA, Gordon A, Fox JC, Chisholm R, Brown B, Regan T, Mooney N, Read SM, Burgman MA (2009) Modelling human impacts on the Tasmanian wedge-tailed eagle (*Aquila audax fleayi*). Biological conservation, 142(11), pp.2438-2448.

Berry KH, Lee JL, Shields TA, Stockton L (2020) The catastrophic decline of tortoises at a fenced natural area. Wildlife Monographs, 205(1), 1-53.

Biasotto LD, Pacífico EC, Paschotto FR, Filadelfo T, Couto MB, Sousa AEB, Mantovani P, Silveira LF, Ascensão F, Tella JL, Kindel A (2023) Power line electrocution as an overlooked threat to Lear's Macaw (*Anodorhynchus leari*). Ibis, 165(3), pp.998-1006.

Bisson IA, Ferrer M, Bird DM (2002) Factors influencing nest-site selection by Spanish Imperial Eagles. Journal of Field Ornithology, 73(3), 298-302.

Blasdell KR, McNamara B, O'Brien DP, Tachedijian M, Boyd V, Dunn M, Mee PT, Clayton S, Gaburro J, Smith I, Gibney KB, Tay EL, Hobbs EC, Waidyatillake N, Lynch SE, Stinear TP, Athan E (2022) Environmental risk factors associated with the presence of *Mycobacterium ulcerans* in Victoria, Australia. PLoS ONE, 17(9 September), e0274627.

Bohall-Wood P, Bosworth SB, Dettmers R (2006) Cerulean Warbler Abundance and Occurrence Relative to Large-Scale Edge and Habitat Characteristics. The Condor, 108(1), 154-165.

Boshoff AF, Minnie JC, Tambling CJ, Michael MD (2011) The impact of power line-related mortality on the Cape Vulture *Gyps coprotheres* in a part of its range, with an emphasis on electrocution. Bird Conservation International, 21(3), 311-327.

Bradley HS, Craig MD, Tomlinson S, Cross AT, Bamford MJ, Bateman PW (2023) Ecological Considerations When Designing Mitigation Translocations: An Australian Reptile Case Study. Animals, 13(16) 2594.

Brand CJ (2013) Wildlife mortality investigation and disease research: contributions of the USGS National Wildlife Health Center to endangered species management and recovery. Ecohealth, 10(4), 446-454.

Braun CE (1998) Sage grouse declines in western North America: what are the problems. In Proceedings of the Western Association of State Fish and Wildlife Agencies (Vol. 78).

Bromfield M (2019) Power line-related mortalities of the Cape Vulture (Gyps coprotheres) within the Eastern Cape, South Africa. Doctoral dissertation, Faculty of Science, University of the Witwatersrand, Johannesburg.

Brush T (2009) Range Expansions and New Breeding Records of Birds in Tamaulipas, Mexico. The Southwestern Naturalist, 54(1), 91-96.

Burdett EM, Muriel R, Morandini V, Kolnegari M, Ferrer M (2022). Power lines and birds: drivers of conflict-prone use of pylons by nesting white storks (Ciconia ciconia). Diversity, 14(11), 984.

Burnside RJ, Collar NJ, Dolman PM (2018) Dataset on the numbers and proportion of mortality attributable to hunting, trapping, and powerlines in wild and captive-bred migratory Asian houbara *Chlamydotis macqueenii*. Biological Conservation, 28, 357-366.

Burnside R, Collar N, Koshkin M, Dolman P (2015) Avian powerline mortalities, including Asian Houbaras *Chlamydotis macqueenii*, on the Central Asian flyway in Uzbekistan. Sandgrouse, 37, 161-168.

Byju, H, Raveendran N, Mathiyazhagan AJ (2023) Powerline pylons: an unusual nesting success of White-bellied Sea-Eagle *Haliaeetus leucogaster* (Gmelin, 1788)(Aves: *Accipitriformes: Accipitridae*) from Ramanathapuram, southeastern coast of India. Journal of Threatened Taxa, 15(7), 23610-23614.

Caizergues A, Ellison LN (1997) Survival of black grouse Tetrao tetrix in the French Alps. Wildlife Biology, 3(3-4), 177-186.

Canfield JE (1984) Elk habitat use and the impact of the construction and energization of a 500-KV AC powerline on the North Boulder winter range, Montana. Doctoral dissertation, Montana State University-Bozeman, College of Letters & Science.

Cecala KK, Lowe WH, Maerz JC (2014) Riparian disturbance restricts in-stream movement of salamanders. Freshwater Biology, 59(11), 2354-2364.

Chandler J, Clauss B, Olsen G (2014) A review of parent-rearing Whooping Cranes at Patuxent Wildlife Research Center, 1988-2003. Thirteenth North American Crane Workshop, 118.

Chevallier C, Hernández‐Matías A, Real J, Vincent‐Martin N, Ravayrol A, Besnard A (2015) Retrofitting of power lines effectively reduces mortality by electrocution in large birds: an example with the endangered Bonelli's eagle. Journal of Applied Ecology, 52(6), 1465-1473.

Clancy GP (2010) Causes of Mortality in the Black-necked Stork *Ephippiorhynchus asiaticus australis* in New South Wales. Australian Field Ornithology, 27(2), 65-75.

Clancy GP (2011) The feeding behaviour and diet of the Black-necked Stork *Ephippiorhynchus asiaticus australis* in northern New South Wales. Corella, 36(1), 17.

Clarke DJ, White JG (2008) Management of power line easement vegetation for small mammal conservation in Australia: a case study of the broad-toothed rat (*Mastacomys fuscus).* The Eighth International Symposium on Environmental Concerns in Rights-of-Way Management.

Cole SG, Dahl EL (2013) Compensating white-tailed eagle mortality at the Smøla wind-power plant using electrocution prevention measures. Wildlife Society Bulletin, 37(1), 84-93.

Collar NJ, Wacher T (2023) The conservation status of the Nubian Bustard *Nubotis nuba*: a review and prognosis. Bird Conservation International, 33, e76.

Colman JE, Bergmo T, Tsegaye D, Flydal K, Eftestøl S, Lilleeng MS, Moe SR (2017) Wildlife response to infrastructure: the problem with confounding factors. Polar Biology, 40, 477-482.

Colman JE, Tsegaye D, Flydal K, Rivrud IM, Reimers E, Eftestøl S (2015) High-voltage power lines near wild reindeer calving areas. European Journal of Wildlife Research, 61, 881-893.

Cooper BA, Day RH (1998) Summer behavior and mortality of Dark-rumped Petrels and Newell's Shearwaters at power lines on Kauai. Colonial Waterbirds, 11-19.

Corrêa FM, Chaves ÓM, Printes RC, Romanowski HP (2018) Surviving in the urban–rural interface: Feeding and ranging behavior of brown howlers (*Alouatta guariba clamitans*) in an urban fragment in southern Brazil. American Journal of Primatology, 80(6), e22865.

Crivelli AJ, Jerrentrup H, Mitchev T (1988) Electric power lines: a cause of mortality in *Pelecanus crispus* Bruch, a world endangered bird species, in Porto-Lago, Greece. Colonial Waterbirds, 301-305.

Cunningham SJ, Madden CF, Barnard P, Amar A (2016) Electric crows: powerlines, climate change and the emergence of a native invader. Diversity and Distributions, 22(1), 17-29.

Dawe DA, Parisien MA, Boulanger Y, Boucher J, Beauchemin A, Arseneault D (2022) Short‐ and long‐term wildfire threat when adapting infrastructure for wildlife conservation in the boreal forest. Ecological Applications, 32(6), 1-18.

Day RH, Cooper BA (2022) Behavior of Hawaiian Petrels and Newell's Shearwaters (Aves: Procellariiformes) Around Electrical-Transmission Lines on Kaua‘i Island, Hawaiian Islands. Pacific Science, 76(1), 53-67.

De Oliveira SM, Murray PJ, De Villiers DL, Baxter GS (2014) Ecology and movement of urban koalas adjacent to linear infrastructure in coastal south-east Queensland. Australian Mammalogy, 36(1), 45-54.

Dell DA, Zwank PJ (1986) Impact of a high-voltage transmission line on a nesting pair of Southern Bald Eagles in Southeast Louisiana. Journal of Raptor Research, 20(3), 5.

Dell'Omo G, Costantini D, Lucini V, Antonucci G, Nonno R, Polichetti A (2009) Magnetic fields produced by power lines do not affect growth, serum melatonin, leukocytes and fledging success in wild kestrels. Comparative Biochemistry and Physiology Part C: Toxicology & Pharmacology, 150(3), 372-376.

Desmecht B (2017) Impacts of power lines on brown bear movement in central Sweden. Université de Liège, Liège, Belgique. Gembloux Agro-Bio Tech, département de gestion des ressources forestières, Norwegian University of Life Sciences (NMBU).

Dias A, Palma L, Carvalho F, Neto D, Real J, Beja P (2017) The role of conservative versus innovative nesting behavior on the 25-year population expansion of an avian predator. Ecology & Evolution, 7(12), 4241-4253.

Dixon A, Batbayar N, Bold B, Davaasuren B, Erdenechimeg T, Galtbalt B, Tsolmonjav P, Ichinkhorloo S, Gunga A, Purevochir G, Rahman ML (2020) Variation in electrocution rate and demographic composition of Saker Falcons electrocuted at power lines in Mongolia. Journal of Raptor Research, 54(2), 136-146.

Dolman P M, Scotland KM, Burnside RJ, Collar NJ (2021). Sustainable hunting and the conservation of the threatened houbara bustards. Journal for Nature Conservation, 61, 126000.

Donázar JA, Palacios CJ, Gangoso L, Ceballos O, González MJ, Hiraldo F (2002) Conservation status and limiting factors in the endangered population of Egyptian vulture (*Neophron percnopterus*) in the Canary Islands. Biological Conservation, 107(1), 89-97.

Doucet GJ, Thompson ER (2008) A case of white-tailed deer controlling the woody vegetation in a power line right-of-way located in a winter yard. In Environmental concerns in rights-of-way management: Eighth international symposium, 395-398.

Dunkin SW, Guthery FS, Demaso SJ, Peoples AD, Parry ES (2009) Influence of anthropogenic structures on northern bobwhite space use in western Oklahoma. The Journal of Wildlife Management, 73(2), 253-259.

Dwire AW (2021) Habitat use of wintering Henslow's Sparrows (*Centronyx henslowii)* in power line right-of-ways. Master of Science in Biology, Georgia Southern University, Department of Biology.

Dwyer JF, Doloughan KW (2014) Testing systems of avian perch deterrents on electric power distribution poles in sage-brush habitat. Human-Wildlife Interactions, 8(1), 39-55.

Dwyer JF, Harness RE, Eccleston D (2017) Avian electrocutions on incorrectly retrofitted power poles. Journal of Raptor Research, 51(3), 293-304.

Dwyer JF, Hayes TI, Thorstrom R, Harness RE (2019) Retrofitting power poles to prevent electrocution of translocated Ridgway’s Hawks (*Buteo ridgwayi*). Journal of Caribbean Ornithology, 32, 4-10.

Dwyer JF, Pandey AK, McHale LA, Harness RE (2019) Near-ultraviolet light reduced Sandhill Crane collisions with a power line by 98%. The Condor, 121(2).

Dwyer JF (2006) Electric shock injuries in a Harris's Hawk population. Journal of Raptor Research, 40(3), 193-199.

Eftestøl, S, Tsegaye D, Flydal K, Colman JE (2016) From high voltage (300 kV) to higher voltage (420 kV) power lines: reindeer avoid construction activities. Polar Biology, 39, 689-699.

Ellis KS, Pearse AT, Brandt DA, Bidwell MT, Harrell W, Butler MJ, van der Burg MP (2022) Balancing future renewable energy infrastructure siting and associated habitat loss for migrating whooping cranes. Frontiers in Ecology and Evolution, 10, 931260.

Evans SW (2023) The effects of habitat loss and fragmentation on the relative abundance and conservation of Ludwig's Bustard *Neotis ludwigii* in South Africa. Ostrich, 94(3), 186-203.

Fanke J, Wibbelt G, Krone O (2011) Mortality factors and diseases in free-ranging Eurasian Cranes (*Grus grus*) in Germany. Journal of Wildlife Diseases, 47(3), 627-637.

Fannin, TE (1992) Contaminant Residues in Sandhill Cranes Killed Upon Striking Powerlines in Central Nebraska. Proceedings of the Sixth North American Crane Workshop, 166-70.

Fedy BC, Kirol CP, Sutphin AL, Maechtle TL (2015). The influence of mitigation on sage-grouse habitat selection within an energy development field. PLoS One, 10(4), e0121603.

Fernie KJ, Bird DM (1999) Effects of electromagnetic fields on body mass and food-intake of American kestrels. The Condor, 101(3), 616-621.

Fernie KJ, Bird DM, Dawson RD, Laguë PC (2000) Effects of electromagnetic fields on the reproductive success of American kestrels. Physiological and Biochemical Zoology, 73(1), 60-65.

Ferrer M, Hiraldo F (1991) Evaluation of management techniques for the Spanish imperial eagle. Wildlife Society Bulletin (1973-2006), 19(4), 436-442.

Ferrer M, Hiraldo F (1992) Man-induced sex-biased mortality in the Spanish imperial eagle. Biological Conservation, 60(1), 57-60.

Ferrer M (2001) The Iberian Imperial Eagle, Lynx Nature Books.

Fitrp LL, Ford HA (1997) Status, Habitat and Social Organisation of the Hooded Robin *Melanodryas cucullata* in the New England Region of New South Wales. Australian Bird Watcher, 17(3), 142-155.

Flydal K, Korslund L, Reimers E, Johansen F, Colman JE (2009) Effects of power lines on area use and behaviour of semi-domestic reindeer in enclosures. International Journal of Ecology, 2009.

Folk MJ, Dellinger TA, Leone EH (2013) Is male-biased collision mortality of whooping cranes (*Grus* *americana*) in Florida associated with flock behavior? Waterbirds, 36(2), 214-219.

Folk MJ, SA Nesbitt, JM Parker, MG Spalding, SB Baynes, KL Candelora (2008) Current status of nonmigratory whooping cranes in Florida. In: Folk, MJ and SA Nesbitt, eds. Proceedings of the Tenth North American Crane Workshop, Feb. 7-10, 2006, Zacatecas City, Zacatecas, Mexico: North American Crane Working Group, 7-12.

Fox NC, Wynn C (2010) The impact of electrocution on the New Zealand falcon (*Falco novaeseelandiae*). Notornis, 57(2), 71-74.

Fritz J, Kramer R, Hoffmann W, Trobe D, Unsold M (2017) Back into the wild: establishing a migratory Northern bald ibis *Geronticus eremita* population in Europe. International Zoo Yearbook, 51(1), 107-123.

Frost D (2008) The use of ‘flight diverters’ reduces mute swan *Cygnus olor* collision with power lines at Abberton Reservoir, Essex, England. Conservation Evidence, 5, 83-91.

Gahbauer MA, Bird DM, Clark KE, French T, Brauning DW, Mcmorris FA (2015) Productivity, mortality, and management of urban peregrine falcons in northeastern North America. The Journal of Wildlife Management, 79(1), 10-19.

Galmes MA, Sarasola JH, Grande JM, Vargas FH (2018) Electrocution risk for the endangered Crowned Solitary Eagle and other birds in semiarid landscapes of central Argentina. Bird Conservation International, 28(3), 403-415.

Gangoso L, Palacios CJ (2002) Endangered Egyptian Vulture (*Neophron percnopterus*) entangled in a power line ground-wire stabilizer. Journal of Raptor Research, 36(3), 238-239.

Garrido JR, Fernández-Cruz M.(2003). Effects of power lines on a White Stork *Ciconia ciconia* population in central Spain. Ardeola, 50(2), 191-200.

Gibson D, Blomberg EJ, Atamian MT, Espinosa SP, Sedinger JS (2018). Effects of power lines on habitat use and demography of greater sage‐grouse (*Centrocercus* *urophasianus*). Wildlife Monographs, 200(1), 1-41.

Gilmer DS, Stewart R (1983). Ferruginous hawk populations and habitat use in North Dakota. The Journal of Wildlife Management, 146-157.

González LM, Margalida A, Manosa S, Sánchez R, Oria J, Molina JI, Caldera J, Aranda A, Prada L, (2007) Causes and spatio-temporal variations of non-natural mortality in the vulnerable Spanish imperial eagle *Aquila adalberti* during a recovery period. Oryx, 41(4), pp.495-502.

Gustin M, Giglio G, Pellegrino SC, Frassanito A, Ferrarini A (2018). Nocturnal flights lead to collision risk with power lines and wind farms in Lesser Kestrels: a preliminary assessment through GPS tracking. Computational Ecology and Software, 8(1), 15-22.

Guyn KL, Clark RG (2000) Nesting Effort of Northern Pintails in Alberta. The Condor, 102(3), 619-628.

Hagen CA, Pitman JC, Loughin TM, Sandercock BK, Robel RJ, Applegate RD (2011) Impacts of anthropogenic features on habitat use by Lesser Prairie-Chickens. In Sandercock, B.K., Martin K, Segelbacher G. Ecology, conservation and management of grouse. Studies in Avian Biology, 39, 63-75.

Hagen CA, Pitman JC, Sandercock BK, Robel RJ, Applegate RD (2007) Age-Specific Survival and Probable Causes of Mortality in Female Lesser Prairie-Chickens. Journal of Wildlife Management, 71(2), 518-525.

Hansen EP, Stewart AC, Frey SN (2016). Influence of transmission line construction on winter sage-grouse habitat use in southern Utah. Human-Wildlife Interactions, 10(2), 169-187.

Harness RE (2004) Bald Eagle (*Haliaeetus leucocephalus*) electrocutions and artificial food sources in Alaska. In Environment Concerns in Rights-of-Way Management 8th International Symposium, 12-16.

Hausleitner D, Wallace J (2012) Common Nighthawk Use of Transmission Lines in the Waneta Expansion Project Area. Seepanee Ecological Consulting.

Hays QR, Tredennick AT, Carlisle JD, Collins DP, Carleton SA (2021) Spatially Explicit Assessment of Sandhill Crane Exposure to Potential Transmission Line Collision Risk. The Journal of Wildlife Management, 85(7), 1440-1449.

Hellmich J, Idaghdour Y (2002) The Great Bustard Otis tarda population in Morocco 1998-2001. Bird Conservation International, 12(1), 19-23.

Henderson IG, Langston RH, Clark NA (1996). The response of common terns Sterna hirundo o to power lines: an assessment of risk in relation to breeding commitment, age and wind speed. Biological Conservation, 77(2-3), 185-192.

Hernandez-Matias A, Real J, Pares F, Pradel R (2015) Electrocution threatens the viability of populations of the endangered Bonelli's eagle (*Aquila fasciata*) in Southern Europe. Biological Conservation, 191, 110-116.

Hunter EA, Dwire AW, Schneider TM (2022). Demography and site fidelity of a grassland bird, the Henslow's Sparrow, in powerline right-of-way habitat. Journal of Field Ornithology, 93 (1), 9.

Husby M, Pearson M (2022) Wind farms and power lines have negative effects on territory occupancy in Eurasian eagle owls (*Bubo bubo*). Animals, 12(9), 1089.

Ivey G (2014) A model for mitigating loss of cranes from power line collisions in Aborn DA, Urbanek RP Proceedings of the North American Crane Workshop 14-17 April 2014, Lafayette, Louisiana.

Jackson SM, Parsons M, Baseler M, Stanton D (2020) Landscape management of the mahogany glider (*Petaurus gracilis*) across its distribution: Subpopulations and corridor priorities. Australian Mammalogy, 42(2), 152-159.

Janiszewski T, Minias P, Wojciechowski Z (2015) Selective Forces Responsible for Transition to Nesting on Electricity Poles in the White Stork *Ciconia ciconia*. Ardea, 103(1), 39-50.

Janss GF, Ferrer M (2000). Common crane and great bustard collision with power lines: collision rate and risk exposure. Wildlife Society Bulletin, 675-680.

Janss GF, Ferrer M (2001) Avian electrocution mortality in relation to pole design and adjacent habitat in Spain. Bird Conservation International, 11(1), 3-12.

Jenkins AR, Shaw JM, Smallie JJ, Gibbons B, Visagie R, Ryan PG (2011) Estimating the impacts of power line collisions on Ludwig’s Bustards *Neotis ludwigii*. Bird Conservation International, 21(3), 303-310.

Jha RR, Thakuri JJ, Rahmani AR, Dhakal M, Khongsai N, Pradhan NMB, Shinde N, Chauhan BK, Talegaonkar RK, Barber IP, Buchanan, GM (2018) Distribution, movements, and survival of the critically endangered Bengal Florican *Houbaropsis bengalensis* in India and Nepal. Journal of Ornithology, 159, 851-866.

Jodice PGR, Epperson DM, HenkVisser G (2006) Daily Energy Expenditure in Free-ranging Gopher Tortoises (*Gopherus Polyphem*us) Copeia, 1, 129-136.

Kaiser SA, Lindell, CA (2007) Effects of distance to edge and edge type on nestling growth and nest survival in the Wood Thrush. The Condor, 109(2), 288-303.

Kalpakis S, Mazaris AD, Mamakis Y, Poulopoulos Y (2009) A retrospective study of mortality and morbidity factors for Common Buzzards *Buteo buteo* and Long-legged *Buzzards Buteo rufinus* in Greece: 1996-2005. Bird Conservation International, 19(1), 15-21.

Kaługa I, Sparks TH, Tryjanowski, P (2011) Reducing death by electrocution of the white stork *Ciconia ciconia*. Conservation Letters, 4(6), 483-487.

Kambouris PJ, Kavanagh RP, Rowley KA (2013) Distribution, habitat preferences and management of the yellow-bellied glider, *Petaurus australis*, on the Bago Plateau, New South Wales: a reassessment of the population. Wildlife Research, 40, 599-614.

Kelly A, Kelly S (2005) Are mute swans with elevated blood lead levels more likely to collide with overhead power lines? Waterbirds, 28(3), 331-334.

Kirol CP, Sutphin AL, Bond L, Fuller MR, Maechtle TL (2015) Mitigation effectiveness for improving nesting success of greater sage‐grouse influenced by energy development. Wildlife Biology, 21(2), 98-109.

Klaus NA, Buehler DA (2001) Golden-winged Warbler breeding habitat characteristics and nest success in clearcuts in the Southern Appalachian Mountains. The Wilson Bulletin, 113(3), 297-301.

Kochert MN, Steenhof K, Brown JL (2019) Effects of Nest Exposure and Spring Temperatures on Golden Eagle Brood Survival: An Opportunity for Mitigation. Journal of Raptor Research, 53(1), 91-97.

Kohl MT, Messmer TA, Crabb BA, Guttery MR, Dahlgren DK, Larsen RT, Frey SN, Liguori S, Baxter, RJ (2019) The effects of electric power lines on the breeding ecology of greater sage-grouse. PLoS One, 14(1), p.e0209968.

Kolnegari M, Basiri AA, Hazrati M, Dwyer JF (2020) Effects of Nest Box Installation on a Distribution Power Line: Increased Eurasian Kestrel Nesting, Reduced Electrocutions, and Reduced Electrical Faults. Journal of Raptor Research, 54(4), 431-439.

Kotoane M (2004) Modelling risk of Blue Crane (*Anthropoides paradiseus*) collision with power lines in the Overberg region. Doctoral dissertation, Stellenbosch: Stellenbosch University.

Krüger SC, Amar A (2021) The Ecology and Management of a Critically Endangered Population of Bearded Vultures. The Encyclopedia of Conservation, Elsevier, 2022, 313-323.

Kruger SC, Amar A (2017) Productivity of the declining Bearded Vulture *Gypaetus barbatus* population in southern Africa. Ostrich, 88, 139-145.

Kurhade S (2017) Greater Flamingo Phoenicopterus roseus mortality due to electrocution, in Ahmednagar District, Maharashtra, India. Wildlife Society Bulletin, 29(3), 804-813.

Lane SJ, Alonso JC, Martin CA (2001) Habitat preferences of great bustard Otis tarda flocks in the arable steppes of central Spain: are potentially suitable areas unoccupied? Journal of Applied Ecology, 38(1), 193.

Ledger JA, Annegarn HJ (1981) Electrocution hazards to the cape vulture *Gyps coprotheres* in South Africa. Biological Conservation, 20(1), 15-24.

Lehnen SE, Rodewald AD (2013) Daily and seasonal movements of a shrubland-obligate breeder in relation to mature forest edge habitat. Forest Ecology and Management, 305, 112-119.

Litzgus JD, Mousseau TA (2004) Home Range and Seasonal Activity of Southern Spotted Turtles (*Clemmys guttata*): Implications for Management. Copeia, 4, 804-817.

Litzgus JD, Mousseau TA (2006) Geographic Variation in Reproduction in a Freshwater Turtle (*Clemmys Guttata*). Herpetologica, 62(2), 132-140.

López-López P, Ferrer M, Madero A, Casado E, McGrady M (2011). Solving man-induced large-scale conservation problems: the Spanish Imperial Eagle and power lines. PloS one, 6(3), e17196.

Lopez-Peinado A, Singh NJ, Urios V, Lopez-Lopez P (2023) Experimental food subsidies keep eagles inside protected areas: implications for conservation and resource management. Biological Conservation, 286, 110259.

Lóránt M, Vadász C (2014) The Effect of Above-Ground Medium Voltage Power Lines on Displaying Site Selection of the Great Bustard in Central Hungary. Ornis Hungarica, 22(2), 42-49.

Loudon JE, Howells ME, Wolfe CA, Buana IN, Buda W, Wandia IN, Gusti Agung Arta Putra I, Patterson M, Fuestes A (2024) Healing Hanuman’s Army: Veterinary Care as a Core Component of One Health Principles in a Southeast Asian Monkey Forest. Animals 14(1), 117.

Lucas PDS, Alves-Eigenheer M, Francisco TM, Dietz JM, Ruiz-Miranda CR (2019) Spatial response to linear infrastructures by the endangered golden lion tamarin. Diversity, 11(7), 100.

Lutkens R, Eder F (1977) On the fate of peripheral populations of the European Bustard (*Otis tarda, L.*) in Niederösterreich (Lower Austria). Journal of Ornithology, 118(1), 93-105.

Mahood SP, Silva JP, Dolman PM, Burnside RJ (2018) Proposed power transmission lines in Cambodia constitute a significant new threat to the largest population of the Critically Endangered Bengal florican *Houbaropsis bengalensis*. Oryx, 52(1), 147-155.

Mahood SP, Sum P, Hong C, Son V, Gnuen G, Ly R, Tizard R (2022) A new power transmission line causes significant mortality in the largest remaining population of Critically Endangered Bengal floricans *Houbaropsis bengalensis* in Cambodia. Cambodian Journal of Natural History, 84.

Mali S, Soni B, Bora N, Narwade S (2023) High Congregation of Demoiselle Cranes at a Wintering Site in Khichan, Rajasthan, India. Journal of the Bombay Natural History Society, 120.

Malovichko LV, Poddubnaya NY, Kulakov DV (2023) Ecology and reproductive biology of the European Roller *Coracias garrulus L*., 1758 in Stavropol Region. Ecosystem Transformation, 6(1), 51-71.

Manosa S, Real J (2001). Potential negative effects of collisions with transmission lines on a Bonelli's Eagle population. Journal of Raptor Research, 35(3), 10.

Manosa S, Bota G (2023) Modelling the effectivity of a land sparing strategy to preserve an endangered steppe-land bird population in cereal farmland: Scopes and limits. Biological Conservation, 288, 110386.

Marcelino J, Moreira F, Manosa S, Cusco F, Morales MB, De La Morena ELG, Bota G, Palmeirim JM, Silva, J. P (2018) Tracking data of the Little Bustard *Tetrax tetrax* in Iberia shows high anthropogenic mortality. Bird Conservation International, 28(4), 509-520.

Marcelino J, Moreira F, Franco AMA, Soriano-Redondo A, Acacio M, Gauld J, Castro Rego F, Paulo Silva J, Catry I (2021) Flight altitudes of a soaring bird suggest landfill sites as power line collision hotspots. Journal of Environmental Management, 294, 113149.

Margalida A, Heredia R, Razin M, Hernández M (2008) Sources of variation in mortality of the Bearded Vulture *Gypaetus barbatus* in Europe. Bird Conservation International, 18(1), 1-10.

Marques AT, Moreira F, Alcazar R, Delgado A, Godinho C, Sampaio H, Rocha P, Sequeira N, Palmeirim JM, Silva, JP (2020) Changes in grassland management and linear infrastructures associated to the decline of an endangered bird population. Scientific Reports, 10(1), 15150.

Marques AT, Palma L, Lourenço R, Cangarato R, Leitão, A, Mascarenhas M, Tiago Tavares J, Tome R, Moreira F, Beja P (2022) Individual variability in space use near power lines by a long‐lived territorial raptor. Ecology and Evolution, 12(4), e8811.

Martens FR, Pfeiffer MB, Downs CT, Venter JA (2018) Post-fledging movement and spatial ecology of the endangered Cape Vulture (*Gyps coprotheres*). Journal of Ornithology, 159, 913-922.

Martinez JA, Martinez JE, Manosa S, Zuberogoitia I, Calvo JF (2006). How to manage human-induced mortality in the Eagle Owl *Bubo bubo*. Bird Conservation International, 16(3), 265-278.

Martín, CA , Alonso, JC, Alonso JA, Palacín C, Magana M, Martín B (2007) Sex‐biased juvenile survival in a bird with extreme size dimorphism, the great bustard Otis tarda. Journal of Avian Biology, 38(3), 335-346.

McGrady MJ, Ross, S, AlJahdhami MH, Meyburg BU (2024) Characterising ecological resource utilization by the Endangered Egyptian vulture in Oman to better manage and mitigate electrocution risk. Journal for Nature Conservation, 126565.

Messmer TA, Hasenyager R, Burruss J, Liguori S (2013) Stakeholder contemporary knowledge needs regarding the potential effects of tall structures on sage-grouse. Human-Wildlife Interactions, 7(2), 273-298.

Mikkola H, Tornberg R (2014) Sex-specific diet analysis of the Eurasian Eagle Owl in Finland. Ornis Fennica, 91(3), 195.

Miller JL, Spalding MG, Folk MJ (2010) Leg problems and power line interactions in the Florida resident flock of whooping cranes. In: Hartup, Barry K., ed., Proceedings of the Eleventh North American Crane Workshop, Sep 23-27, 2008, Wisconsin Dells, Wisconsin (Baraboo, WI: North American Crane Working Group, 2010), 156-165.

Mo M, Coutts-McClelland K, Wilson V, Haering R, Oliver L, Bell L, Lunney D (2023) Managing the Grey-headed Flying-fox as a threatened species in New South Wales two decades on: threats and conservation issues. Australian Zoologist, 42(4), 897-918.

Mojica EK, Dwyer JF, Harness RE, Williams, GE, Woodbridge B (2018) Review and synthesis of research investigating golden eagle electrocutions. The Journal of Wildlife Management, 82(3), 495-506.

Mojica EK, Watts BD, Paul JT, Voss ST, Pottie J (2009) Factors contributing to Bald Eagle electrocutions and line collisions on Aberdeen Proving Ground, Maryland. Journal of Raptor Research, 43(1), 57-61.

Monticelli C, Maciel PC, de Oliveira Garcia F (2022) Rope bridges provide safe connectivity for the southern brown howler monkey (*Alouatta guariba clamitans* Cabrera, 1940) in an urban Atlantic Forest remnant. Folia Primatologica, 93(3-6), 519-532.

Morales, MB, Bretagnolle V (2022) An update on the conservation status of the Little Bustard *Tetrax tetrax* : global and local population estimates, trends, and threats. Bird Conservation International, 32(3), 337-359.

Morales MB, Bretagnolle V, Arroyo B (2005) Viability of the Endangered Little Bustard *Tetrax tetrax* Population of Western France. Biodiversity & Conservation, 14(13), 3135-3150.

Moreira F, Encarnação V, Rosa G, Gilbert N, Infante S, Costa J, D’Amico M, Martins RC, Catry I (2017) Wired: impacts of increasing power line use by a growing bird population. Environmental Research Letters, 12(2), 024019.

Moreira F, Martins RC, Catry I, D'Amico M (2018) Drivers of power line use by white storks: A case study of birds nesting on anthropogenic structures. Journal of Applied Ecology, 55(5), 2263-2273.

Morkill AE (1990) Effectiveness of markers in reducing Sandhill Crane collisions with powerlines. Masters Thesis, University of Wyoming.

Morkill AE, Anderson SH (1991) Effectiveness of marking powerlines to reduce Sandhill Crane collisions. Wildlife Society Bulletin (1973-2006), 19(4), 442-449.

Morrow L, Morrow J (2018) American Kestrel Surviving with Electric Shock Injuries. Journal of Raptor Research, 52(1), 102-103.

Murn C, Botha A, Wilson B (2017) The Changing Sizes of Critically Endangered White-Backed Vulture Breeding Colonies Around Kimberley, South Africa. African Journal of Wildlife Research, 47(2), 144-148.

Murphy N K, Boudreau MR, Dorr BS, Slankard K, Rush SA (2024) Identifying priority mitigation areas for human–osprey conflict. The Journal of Wildlife Management, e22540.

Murphy RK, Dwyer JF, Mojica EK, McPherron MM, Harness RE (2016) Reactions of Sandhill Cranes approaching a marked transmission power line. Journal of Fish and Wildlife Management, 7(2), 480-489.

Murphy RK, Mojica EK, Dwyer JF, McPherron MM, Wright GD, Harness RE, Pandey AK, Serbousek KL (2016) Crippling and nocturnal biases in a study of Sandhill Crane (*Grus canadensis*) collisions with a transmission line. Waterbirds, 39(3), 312-317.

Naugle DE, Walker BL, Doherty KE (2006) Sage-grouse population response to coal-bed natural gas development in the Powder River Basin: interim progress report on region-wide lek-count analyses. University of Montana, Wildlife Biology Program, College of Forestry and Conservation.

Nellemann C, Vistnes I, Jordhøy P, Strand O (2001) Winter distribution of wild reindeer in relation to power lines, roads and resorts. Biological conservation, 101(3), 351-360.

Neumann W, Ericsson G, Dettki H, Radeloff VC (2013) Behavioural response to infrastructure of wildlife adapted to natural disturbances. Landscape and Urban Planning, 114, 9-27.

Newman JR, Newman CM, Lindsay JR, Merchant B, Avery ML, Pruett-Jones S (2008) Monk parakeets: an expanding problem on power lines and other electrical utility structures. In Environment concerns rights-of-way management 8th international symposium (pp. 355-363).

Nygård T, Jacobsen KO, Gjershaug J O (2023) Home-range, movements and use of powerline poles of Eagle-Owls (*Bubo* *bubo*) at an island population in northern Norway. Ornis Fennica. 2023, 100, 99-111.

Oppel S, Arkumarev V, Bakari S, Dobrev V, Saravia-Mullin V, Adefolu S, Sozuer LA, Apeverga PT, Arslan S, Barshep Y, Bino T, Bounas A, Cetin T, Dayyoub M, Dobrev D, Duro K, Moghrabi LE, ElSafoury H, Endris A, Asswad NG, Nikolov SC (2021) Major threats to a migratory raptor vary geographically along the eastern Mediterranean flyway. Biological Conservation, 262, 109277.

Ossenkopp KP, Kavaliers M, Lipa S (1990) Increased mortality in land snails (*Cepaea nemoralis*) exposed to powerline (60-Hz) magnetic fields and effects of the light-dark cycle. Neuroscience letters, 114(1), 89-94.

Ott JP, Hanberry BB, Khalil M, Paschke MW, Van Der Burg MP, Prenni AJ (2021) Energy development and production in the Great Plains: Implications and mitigation opportunities. Rangeland Ecology & Management, 78, 257-272.

Özgencil İK, Akarsu F, Karataş, MM, Gursoy-Egen A, Saygili-Yigit FA, Karakaya M, Soyluer M (2022). Current status of Great Bustard *Otis tarda* in Turkey: population size, distribution, movements, and threats. Bird Conservation International, 32(4), 531-543.

Palacín C, Alonso JC, Martín CA, Alonso JA (2017) Changes in bird‐migration patterns associated with human‐induced mortality. Conservation Biology, 31(1), 106-115.

Palacin C, Fairas I, Alonso JC (2023) Detailed mapping of protected species distribution, an essential tool for renewable energy planning in agroecosystems. Biological Conservation, 277, 109857.

Palei NC, Palei HS, Rath BP, Kar CS (2014) Mortality of the Endangered Asian elephant *Elephas* *maximus* by electrocution in Odisha, India. Oryx, 48(4), 602-604.

Palei NC, Rath BP, Pradhan SD, Mishra AK (2015. An Assessment of Human Elephant (*Elephas maximus*) Conflict (HEC) in Mahanadi Elephant Reserve and Suggested Measures for Mitigation, Odisha, India. Middle-East J. Sci. Res, 23, 1824-1831.

Paquet PC, Callahan C (1996) Effects of linear developments on winter movements of gray wolves in the Bow River Valley of Banff National Park, Alberta. US Department of Transportation,

Parayko NW, Ng JW, Marley J, Wolach RS, Wellicome TI, Bayne EM (2021) Response of Ferruginous Hawks to temporary habitat alterations for energy development in southwestern Alberta. Avian Conservation and Ecology 16(2), 17.

Patten MA, Barnard AA, Curry CM, Dang H, Loraamm RW (2021) Forging a Bayesian link between habitat selection and avoidance behavior in a grassland grouse. Scientific Reports, 11(1), 2791.

Pérez-García JM, Sebastián-González E, Botella F, Sánchez-Zapata JA (2016). Selecting indicator species of infrastructure impacts using network analysis and biological traits: bird electrocution and power lines. Ecological indicators, 60, 428-433.

Peterson JM, Earl JE, Fuhlendorf SD, Elmore DR, Haukos DA, Tanner AM, Carleton SA (2020) Estimating response distances of lesser prairie‐chickens to anthropogenic features during long‐distance movements. Ecosphere, 11(9), 1-15.

Phipps WL, Wolter K, Michael MD, MacTavish LM, Yarnell RW (2013) Do power lines and protected areas present a catch-22 situation for cape vultures (*Gyps* *coprotheres*)?. PLoS One, 8(10), e76794.

Picardi S, Messmer T, Crabb B, Kohl M, Dahlgren D, Frey N, Larsen R, Baxter R (2020) Predicting greater sage‐grouse habitat selection at the southern periphery of their range. Ecology & Evolution, 10(23), 13451, 13463.

Plumb RT, Lautenbach JM, Robinson SG, Haukos DA, Winder VL, Hagen CA, Sullins DS, Pitman JC, Dahlgren DK (2019) Lesser prairie‐chicken space use in relation to anthropogenic structures. The Journal of Wildlife Management, 83(1), 216-230.

Plummer MV (2002) Observations on hibernacula and overwintering ecology of Eastern hog-nosed snakes (*Heterodon platirhinos*). Herpetological Review, 33(2), 89.

Podolsky R, Ainley DG, Spencer G, Deforest L, Nur N (1998) Mortality of Newell's Shearwaters caused by collisions with urban structures on Kauai. Colonial Waterbirds, 20-34.

Pretorius MD, Galloway-Griesel TL, Leeuwner L, Michael MD, Durgapersad K, Chetty, K (2023) Defining Collision Risk: Lesser Flamingo *Phoeniconaias minor* Power Line Collision Sensitivity and Exposure for Proactive Mitigation. Birds, 4(4), 315-329.

Pretorius MD, Leeuwner L, Tate GJ, Botha A, Michael MD, Durgapersad, K, Chetty, K (2020) Movement patterns of lesser flamingos *Phoeniconaias minor*: nomadism or partial migration? Wildlife Biology, 2020(3), 1-11.

Pruett CL, Patten MA, Wolfe DH (2009) Avoidance behavior by prairie grouse: implications for development of wind energy. Conservation Biology, 23(5), 1253-1259.

Puzović S (2008) Nest occupation and prey grabbing by Saker Falcon (*Falco* *cherrug*) on power lines in the province of Vojvodina (Serbia). Archives of Biological Sciences, 60(2), 271-277.

Raab R, Julius E, Spakovszky P, Nagy S (2009) Guidelines for best practice on mitigating impacts of infrastructure development and afforestation on the Great Bustard. In Prepared for the Memorandum of Understanding on the conservation and management of the Middle-European population of the Great Bustard under the Convention on Migratory species (CMS). Birdlife International. European Division.

Raab R, Spakovszky P, Julius E, Schuetz C, Schulze CH (2011) Effects of power lines on flight behaviour of the West-Pannonian Great Bustard *Otis tarda* population. Bird Conservation International, 21(2), 142-155.

Raine AF, Holmes ND, Travers M, Cooper BA, Day RH (2017) Declining population trends of Hawaiian petrel and newell’s shearwater on the island of kaua’i (Hawaii, USA: The Condor) 119 (3), 405–415.

Raine AF, Driskill S, Rothe J, Travers MS (2023) Evaluating the breeding phenology of the endangered ‘a ‘o (Newell’s Shearwater *Puffinus newelli*) on Kaua‘i to better focus conservation actions and management decisions. Bird Conservation International, 33, e35.

Ram M, Gadhavi D, Sahu A, Srivastava N, Ali Rather T, Bhatia K, Jhala L, Zala Y, Gadhvi I, Modi V, Jhala D, Patel A, Baraiya S, Devaliya D (2023) Unravelling the secrets of lesser florican: a study of their home range and habitat use in Gujarat, India. Scientific Reports, 13, 19082.

Real J, Grande JM, Mañosa S, Sánchez-Zapata JA (2001) Causes of death in different areas for Bonelli's Eagle *Hieraaetus fasciatus* in Spain. Bird study, 48(2), 221-228.

Reimers E, Dahle B, Eftestøl S, Colman JE, Gaare E (2007) Effects of a power line on migration and range use of wild reindeer. Biological Conservation, 134(4), 484-494.

Reimers E, Eftestøl S, Alemu D T, Granum K (2020) Reindeer fidelity to high quality winter pastures outcompete power line barrier effects. Rangifer, 40(1), 27-40.

Ricard JG, Doucet GJ (1999) Winter use of powerline rights-of-way by moose (*Alces alces*). Alces: A Journal Devoted to the Biology and Management of Moose, 35, 31-40.

Rieucau, G. Vickery WL, Doucet GJ, Laquerre B (2007) An innovative use of white-tailed deer (*Odocoileus* *virginianus*) foraging behaviour in impact studies. Canadian Journal of Zoology, 85(7), 839-846.

Risvoll C, Galafassi D, Veland S, Pavall M, Lifjell T, Lundberg AK, Eilertsen SM (2022) Maps and stories in the creation of richer accounts of change in pastoral landscapes in Nordland, northern Norway. Pastoralism, 12(1), p.45.

Robinson SG (2018) Effects of Landscape Characteristics on Annual Survival of Lesser Prairie-Chickens. The American Midland Naturalist, 180 (1), 66-86.

Roeder DV, Husak MS, Murphy MT, Patten MA (2022) Combined roles for breeding synchrony, habitat and scale as predictors of extrapair paternity. Animal Behaviour, 194, 139-150.

Rojas IA, Gregory T (2022) Canopy bridges: preventing and mitigating anthropogenic impacts on mantled howler monkeys (*Alouatta palliata palliata*) in Costa Rica. Folia Primatologica, 93(3-6), 383-395.

Rollan A, Real J, Bosch R, Tinto A, Hernandez-Matias A (2010) Modelling the risk of collision with power lines in Bonelli’s Eagle *Hieraaetus* *fasciatus* and its conservation implications. Bird Conservation International, 20(3), 279-294.

Rubolini D, Bassi E, Bogliani G, Galeotti P, Garavaglia R (2001) Eagle Owl *Bubo* *bubo* and power line interactions in the Italian Alps. Bird Conservation International, 11(4), 319-324.

Samson A, Kumar PS (2015) Occurrence of Eastern Grass Owl (Tyto longimembris) and the power-line causalities in the Upper Nilgiris, Southern Western Ghats. Newsletter for Birdwatchers, 55, 3.

Santangeli A, Cardillo A, Pes M, Aresu M (2023) Alarming decline of the Little Bustard *Tetrax tetrax* in one of its two population strongholds in Sardinia, Italy/ Bird Conservation International, 33, e57.

Sarasola JH, Galmes MA, Watts BD (2020) Electrocution on power lines is an important threat for the endangered Chaco Eagle (*Buteogallus coronatus*) in Argentina. Journal of Raptor Research, 54(2), 166-171.

Schaub M, Aebischer A, Gimenez O, Berger S, Arlettaz R (2010) Massive immigration balances high anthropogenic mortality in a stable eagle owl population: Lessons for conservation. Biological Conservation, 143(8): 1911-1918.

Senoge ND, Downs CT (2003) The use of cellular telecommunication towers as nesting sites by pied crows (*Corvus albus*) in an urban mosaic landscape. Urban Ecosystems 26(3), 881-892.

Sergio F, Marchesi L, Pedrini P, Ferrer M, Vincenzo P (2004) Electrocution alters the distribution and density of a top predator, the eagle owl *Bubo bubo*. Journal of Applied Ecology 41(5): 836-845.

Sharma HP, Katuwal HB, Regmi S, Suwal RN, Acharya R, Nepali A, Sabin KC, Aryal B, Tamang K, Rawal B, Basnet A, Baral BD, Devkota S, Parajuli S, Regmi N, Kandel P, Subedi B, Giri HS, Kawan S, Thapa GJ, Bhattarai BP (2024) Population and conservation threats to the vulnerable Sarus crane *Grus antigone* in Nepal. Ecology and Evolution, 14(2), e10929.

Shaw JM (2013) Power line collisions in the Karoo conserving Ludwig's bustard. Percy FitzPatrick Institute of African Ornithology, DST/NRF Centre of Excellence, Department of Biological Sciences, Faculty of Science, University of Cape Town,

Shaw JM, Jenkins AR, Smallie JJ, Ryan PG (2010) Modelling power‐line collision risk for the Blue Crane *Anthropoides paradiseus* in South Africa. Ibis, 152(3), 590-599.

Shimada T (2001) Choice of daily flight routes of Greater White-fronted Geese: Effects of power lines. Waterbirds, 425-429.

Shobrak M, Alasmari S, Alqthami A, Alqthami F, Al-Otaibi A, Al Zoubi M, El Moghrabi L, Jbour S, Asswad NG, Oppel, Arkumarev V (2022) Electric infrastructure poses a significant threat at congregation sites of the globally threatened Steppe Eagle *Aquila nipalensis* in Saudi Arabia. Bird Conservation International, 32(2), 313-321.

Shobrak M, Alasmari S, Alqthami A, Alqthami F, Al-Otaibi A, Zoubi M, Nikolov, SC (2020) Congregations and threats of migratory Egyptian Vultures *Neophron* *percnopterus* along the southwest coast of Saudi Arabia. Sandgrouse, 42, 248-258.

Shurtliff QR, Whiting JC (2021) Common raven nesting and spatial distancing on power lines in southeast Idaho, USA. Human–Wildlife Interactions, 15(3), 7.

Silva JP, Arroyo B, Marques AT, Morales MB, Devoucoux P, Mougeot F (2022) Threats affecting Little Bustards: human impacts. In Little Bustard: Ecology and Conservation (pp. 243-271). Cham: Springer International Publishing.

Silva JP, Palmeirim JM, Alcazar R, Correia R, Delgado A, Moreira F (2014) A spatially explicit approach to assess the collision risk between birds and overhead power lines: a case study with the little bustard. Biological Conservation, 170, 256-263.

Silva JP, Santos M, Queirós L, Leitão D, Moreira F, Pinto M, Leqoc M, Cabral JA (2010) Estimating the influence of overhead transmission power lines and landscape context on the density of little bustard *Tetrax tetrax* breeding populations. Ecological Modelling, 221(16), 1954-1963.

Silva JP, Marques AT, Carrapato C, Machado R, Alcazar R, Delgado A, Godinho C, Elias G, Gameiro J (2023) A nationwide collapse of a priority grassland bird related to livestock conversion and intensification. Scientific Reports 13(1), 10005.

Smeraldo S, Bosso L, Fraissinet M, Bordignon L, Brunelli M, Ancillotto L, Russo D (2020) Modelling risks posed by wind turbines and power lines to soaring birds: The black stork (*Ciconia nigra*) in Italy as a case study. Biodiversity and Conservation, 29, 1959-1976.

Smith A, Willebrand T (1999) Mortality causes and survival rates of hunted and unhunted willow grouse. The Journal of wildlife management, 722-730.

Sos-Koroknai V, Solymosi N, Kirko E, Toth T, Marosan M, Sos E (2020) Examination of morbidity and the incidence of electrocution in Common Kestrels (*Falco tinnunculus*) admitted to the Wildlife Rescue Centre at the Budapest Zoo and Botanical Garden between 2014 and 2016. Magyar Allatorvosok Lapja, 142(7), 429–438.

Soutullo A, Lopez-Lopez P, Urios V (2008) Incorporating spatial structure and stochasticity in endangered Bonelli's eagle's population models: Implications for conservation and management. Biological Conservation, 141(4), 1013-1020.

Sovannary S, Sothearen T, Savet E, Virak S, Chhin S (2022). The Effectiveness of Bengal florican (*Houbaropsis bengalensis*) Conservation in Cambodia. Cambodia Journal of Basic and Applied Research, 4(2), 16-37.

St-Amand J, Tremblay JA, Martin K (2021) Stand-level forest management for foraging and nesting of Williamson's sapsuckers. Forest Ecology & Management, 492.

Stehn TV, T Wassenich (2008) Whooping crane collisions with power lines: an issue paper. In: Folk, MJ and SA Nesbitt, Proceedings of the Tenth North American Crane Workshop, Feb. 7-10, 2006, Zacatecas City, Zacatecas, Mexico: North American Crane Working Group, 25-36.

Stiles JH, Jones RH (1998) Distribution of the red imported fire ant, shape *Solenopsis invicta*, in road and powerline habitats. Landscape Ecology, 13, 335-346.

Sundar KSG, Choudhury BC (2005) Mortality of sarus cranes (*Grus antigone*) due to electricity wires in Uttar Pradesh, India. Environmental Conservation, 32(3), 260-269.

Symes CT, Kruger TL (2012) The Persistence of an Apex Avian Predator, Verreauxs' Eagle, *Aquila verreauxii*, in a Rapidly Urbanizing Environment. South African Journal of Wildlife Research, 42(1), 45-53.

Tavecchia G, Adrover J, Navarro AM, Pradel R (2012) Modelling mortality causes in longitudinal data in the presence of tag loss: application to raptor poisoning and electrocution. Journal of Applied Ecology, 49(1), 297-305.

Tella JL, Hernández-Brito D, Blanco G, Hiraldo F (2020) Urban sprawl, food subsidies and power lines: An ecological trap for large frugivorous bats in Sri Lanka? Diversity, 12(3), 94.

Tidemann CR, Nelson JE (2011) Life Expectancy, Causes of Death and Movements of the Grey-Headed Flying-Fox (*Pteropus poliocephalus*) Inferred from Banding. Acta Chiropterologica, 13(2), 419-429.

Tobolka M (2014) Importance of juvenile mortality in Birds' population: early post-fledging mortality and causes of death in white stork *Ciconia ciconia*. Polish Journal of Ecology, 62(4), 807-813.

Todd B D, Rothermel BB, Reed RN, Luhring TM, Schlatter K, Trenkamp L, Gibbons JW (2008) Habitat alteration increases invasive fire ant abundance to the detriment of amphibians and reptiles. Biological Invasions, 10, 539-546.

Tomas G, Barba E, Merino S, Martinez J (2012) Clutch size and egg volume in great tits (*Parus major*) increase under low intensity electromagnetic fields: A long-term field study. Environmental Research, 118, 40-46.

Travers M, Driskill S, Stemen A, Geelhoed T, Golden DM, Koike S, Shipley AA, Moon HE, Anderson T, Bache M, Raine AF (2021) Post-collision impacts, crippling bias, and environmental bias in a study of Newell's Shearwater and Hawaiian Petrel powerline collisions. Avian Conservation & Ecology, 16(1), 1-15.

Tyler NJ, Stokkan KA, Hogg CR, Nellemann C, Vistnes AI (2016) Cryptic impact: Visual detection of corona light and avoidance of power lines by reindeer. Wildlife Society Bulletin, 40(1), 50-58.

Vaitkuvienė D, Dagys M (2014). Possible effects of electromagnetic field on White Storks *Ciconia* *ciconia* breeding on low-voltage electricity line poles. Zoology and Ecology, 24(4), 289-296.

Veltheim I, Cook S, Palmer GC, Hill FR, McCarthy MA (2019). Breeding home range movements of pre-fledged brolga chicks, *Antigone rubicunda* (Gruidae) in Victoria, Australia–Implications for wind farm planning and conservation. Global Ecology and Conservation, 20, e00703.

Viner TC, Kagan RA, Lehner A, Buchweitz JP (2022) Anticoagulant exposure in golden eagle (*Aquila chrysaetos*) power line electrocution and wind turbine mortalities. The Journal of Wildlife Diseases, 58(2), 348-355.

Vistnes I, Nellemann C (2001) Avoidance of cabins, roads, and power lines by reindeer during calving. The Journal of wildlife management, 915-925.

Vistnes I, Nellemann C, Jordhøy P, Strand O (2001) Wild reindeer: impacts of progressive infrastructure development on distribution and range use. Polar biology, 24, 531-537.

Vistnes I, Nellemann C, Jordhøy P, Strand O (2004) Effects of infrastructure on migration and range use of wild reindeer. The Journal of Wildlife Management, 68(1), 101-108.

Walker BL (2008) Greater sage-grouse response to coal-bed natural gas development and West Nile virus in the Powder River Basin, Montana and Wyoming, USA. University of Montana.

Wang, Y, Purev-Ochir G, Gungaa A, Erdenechimeg B, Terbish O, Khurelbaatar D, Wang Z, Mi C, Guo Y (2022) Migration patterns and conservation status of Asian Great Bustard (*Otis tarda dybowskii*) in northeast Asia. Journal of Ornithology, 164, 341-352.

Ward JP, SH Anderson (1992) Sandhill crane collisions with power lines in southcentral Nebraska. In: Wood D. A., ed. 1992. Proceedings 1988 North American Crane Workshop, Feb. 22–24, 1988. Lake Wales, Florida (Tallahassee, FL: State of Florida Game and Fresh Water Fish Commission Nongame Wildlife Program Technical Report #12, 1992), 189-196.

Watts BD, Mojica EK, Paxton BJ (2015) Using Brownian bridges to assess potential interactions between bald eagles and electrical hazards within the upper Chesapeake Bay. The Journal of Wildlife Management, 79(3), 435-445.

Wen-hong D, Guang-mei Z, Gao W (2003) Nesting success of the Meadow Bunting along habitat edges in northeastern China. Journal of Field Ornithology, 74(1), 37-44.

Wiens JD (2017) Spatial Demographic Models to Inform Conservation Planning of Golden Eagles in Renewable Energy Landscapes. Journal of Raptor Research, 51(3), 234-357.

Wiles GJ, Pratt DH, Kastner M, McKinlay G, Chojnacki J, Pendred MM (2020) Distribution, behaviour, and provenance of Oriental Dollarbirds *Eurystomus orientalis* in Micronesia, including the first two records from the Mariana Islands. Bulletin of the British Ornithologists Club 140(1), 85-95.

Wilson RF, Marsh H, Winter J (2007) Importance of canopy connectivity for home range and movements of the rainforest arboreal ringtail possum (*Hemibelideus lemuroides*). Wildlife Research, 34(3), 177-184.

Windingstad RM (1988) Nonhunting mortality in sandhill cranes. The Journal of Wildlife Management, 260-263.

Wolfe DH, Larsson LC, Patten MA (2016) The lesser prairie-chicken in the mixed-grass prairie ecoregion of Oklahoma, Kansas, and Texas. Ecology and Conservation of Lesser Prairie-Chickens, 299–314.

Wolfe DH, Patten MA, Shochat E, Pruett CL, Sherrod SK (2007) Causes and patterns of mortality in lesser prairie-chickens *Tympanuchus pallidicinctus* and implications for management. Wildlife Biology, 13(1), 95-104.

Wood KA, Mitchell C, Griffin L, Hilton GM (2020) Predicting cumulative wind turbine and power line collision mortality for pink-footed geese using an individual-based model. Wildfowl & Wetlands Trust Report, 179.

Young LC, Kohley CR, VanderWerf EA, Fowlke L, Casillas D, Dalton M, Knight M, Pesque A, Dittmar EM, Raine AF, Vynne M (2023) Successful translocation of Newell’s Shearwaters and Hawaiian Petrels to create a new, predator free breeding colony. Frontiers in Conservation Science, 4, 1177789.

Yousefi M, Mohammadi S, Kafash A (2023) Modeling global habitat suitability and environmental predictor of distribution of a Near Threatened avian scavenger at a high spatial resolution. Frontiers in Ecology and Evolution, 11, 1112962.

Grey

Aggarwal M (2019) Saving wildlife from power lines the focus of a new expert panel in Environment Ministry. Firstpost. Available at: <https://www.firstpost.com/tech/science/saving-wildlife-from-power-lines-the-focus-of-a-new-expert-panel-in-environment-ministry-7334541.html>

Aggarwal M (2019) Save wildlife from power lines, says environment ministry’s panel. Mongabay Features. <https://india.mongabay.com/2019/09/save-elephants-and-wildlife-from-power-lines-says-environment-ministrys-panel/>

Audubon (2023) Birds and Clean Energy. Available at: <https://www.audubon.org/our-work/climate/clean-energy>

Arias L (2015) Costa Rica utility company helps protect wildlife along 250 km of power lines. The Tico Times. Available at: <https://ticotimes.net/2015/11/23/ice-improves-costa-rica-wildlife-protection-along-250-km-of-power-lines>

Avian Power Line Interaction Committee (APLIC) (1996) Suggested Practices for Raptor Protection on Power Lines: The State of the Art in 1996. Edison Electric Institute and Raptor Research Foundation. Available at <https://law.resource.org/pub/us/cfr/ibr/002/aplic.raptor.1996.pdf>

Bainbridge B (2021) Birds on a Wire: The Perils of Power Lines. Power Engineers – Member of WSP. Available at: <https://www.powereng.com/library/birds-on-a-wire-the-perils-of-power-lines>

Baziari F (2022) Powerlines as corridors that support biodiversity in suburban landscapes. Conservation Corridor. Conservation Corridor - Corridors in Management, Digests. Available at: <https://conservationcorridor.org/digests/2022/08/powerlines-as-corridors-that-support-biodiversity-in-suburban-landscapes/>

BBC Wildlife (2012) Bill Oddie: Why powerlines aren’t all bad. Available at: <https://www.discoverwildlife.com/animal-facts/birds/bill-oddie-why-powerlines-arent-all-bad>

Bernardino J (2021) What we know (and don't know) about bird collisions with power lines and how to mitigate them. Transport Ecology. Available at: <https://transportecology.info/research/bird-collisions-with-powerlines>

Berkowitz R (2019) Birds Are Dying from Power-Line Collisions—Now There’s a Solution. Scientific American. Available at: <https://www.scientificamerican.com/article/birds-are-dying-from-power-line-collisions-mdash-now-theres-a-solution/>

BirdLife (2007) On the risks to birds from electricity transmission facilities and how to minimise any such adverse effects. BirdLife Birds and Habitats Directive Task Force. Available at: <https://www.cms.int/manage/raptors/sites/default/files/publication/BirdLife__Position_Power_Lines_and_birds_2007_05_10_.pdf>

BirdLife International (2022) New study: How to stop birds from getting electrocuted by and colliding with power lines across the EU. Available at: <https://www.birdlife.org/news/2022/10/24/new-study-how-to-stop-birds-from-getting-electrocuted-by-and-colliding-with-power-lines-across-the-eu/>

BirdLife (2024) Collisions and electrocutions pose real threats for large bodied migratory bird species. Available at: <http://datazone.birdlife.org/sowb/casestudy/collisions-and-electrocutions-pose-real-threats-for-large-bodied-migratory-bird-species>

BirdLife (2024) Reducing the impacts of powerlines on birds. Available at: <http://old.birdlife.org/sowb/casestudy/reducing-the-impacts-of-power-lines-on-birds>

Breyer M (2023) The Main Thing Killing Birds Near Power Lines Isn't Electrocution. TreeHugger. Available at: <https://www.treehugger.com/main-thing-killing-birds-near-power-lines-isnt-electrocution-7569219>.

Carrington D (2014) Animals see power lines as glowing, flashing bands, research reveals. The Guardian. Available at: <https://www.theguardian.com/environment/2014/mar/12/animals-powerlines-sky-wildlife>

Casey M (2017) Land around powerlines could be boon to birds. Available at: <https://phys.org/news/2017-05-powerlines-boon-birds.html#google_vignette>

Cell Press (2023) Illegal shooting kills most birds found dead near power lines. Science Daily. Available at: <https://www.sciencedaily.com/releases/2023/08/230801131630.htm>

Cibio (2023) Launch of LIFE SafeLines4Birds project to reduce mortality of birds along power lines. Available at: <https://www.cibio.up.pt/en/media/launch-of-life-safelines4birds-project-to-reduce-mortality-of-birds-along-power-lines/>

Colley C (2018) Cut-price power lines are killing howler monkeys in Costa Rica. Unearthed -Greenpeace. Available at: <https://unearthed.greenpeace.org/2018/12/19/costa-rica-tourism-howler-monkey/>

Conniff R (2014) Electric Power Rights of Way: A New Frontier for Conservation. Yale Environment360. Available at: <https://e360.yale.edu/features/electric_power_rights_of_way_a_new_frontier_for_conservation>

Cressey D (2014) Why reindeer steer clear of power lines. Nature. Available at: <https://www.nature.com/articles/nature.2014.14868#citeas>

Ecofact (2015) Birds and Powerlines. Available at: <https://ecofact.ie/birds-and-power-lines/>

European Commission – Energy, Climate change, Environment (2023) Power pylons offer pockets of habitat for mammals in intensively farmed landscapes. Available at: <https://environment.ec.europa.eu/news/power-pylons-offer-pockets-habitat-mammals-intensively-farmed-landscapes-2023-08-02_en>

European Commission (2023) Reducing bird mortality caused by power lines. Available at: <https://webgate.ec.europa.eu/life/publicWebsite/project/LIFE21-NAT-FR-LIFE-SAFELINES4BIRDS-101073826/reducing-bird-mortality-caused-by-power-lines>

European Climate, Infrastructure and Environment Executive Agency (2021) Protecting birds from the dangers of power lines. European Union. Available at: <https://cinea.ec.europa.eu/news-events/news/protecting-birds-dangers-power-lines-2021-09-01_en>

Electric and Magnetic Fields Info (2024) Effects on wildlife. Available at: <https://www.emfs.info/research/known-effects/effects-on-wildlife>

Endangered Wildlife Trust (2022) Wildlife and Energy Programme. Available at: <https://ewt.org.za/what-we-do/saving-species/wildlife-and-energy/>

Engler H (2018) Costa Rica Power lines and wildlife electrocutions: A deadly problem. The Tico Times. Available at: <https://ticotimes.net/2018/07/04/power-lines-and-wildlife-electrocutions-a-deadly-problem>

Envass News (2023) The Impacts of Overhead Powerlines of Avifaunal Species. Available at: <https://www.envass.co.za/the-impacts-of-overhead-powerlines-on-avifaunal-species/>

Farnsworth A, Horton KG, Marra PP (2024) To mitigate bird collisions, enforce the Migratory Bird Treaty Act. Available at: <https://www.pnas.org/doi/10.1073/pnas.2320411121>

Foran S (2017) Power lines offer environmental benefits according to study. Available at: <https://phys.org/news/2017-03-power-lines-environmental-benefits.html>

Flynn G, Ball A, Srey V (2022) Opaque infrastructure project ‘a death sentence’ for Cambodia’s Prey Lang Wildlife Sanctuary. Mongabay. Available at: <https://news.mongabay.com/2022/06/opaque-infrastructure-plans-a-death-sentence-for-cambodias-prey-lang-wildlife-sanctuary/>

Gammon K (2019) The Weird Ways Animals Use Roads, Buildings and Power Lines to Their Advantage. AIP Foundation. Available at: <https://www.aip.org/inside-science/the-weird-ways-animals-use-roads-buildings-and-power-lines-to-their-advantage>

Gilad D (2021) So what’s the story with birds and power lines? Norwegian University of Science and Technology (NTNU). Available at: <https://blog.indecol.no/birds-power-lines/>

Greco J (2021) A Shocking Toll - Saving eagles from the lethal hazards of power line electrocution. National Wildlife Federation. Available at: <https://www.nwf.org/Home/Magazines/National-Wildlife/2021/Feb-Mar/Animals/Eagles-and-Powerlines>

Haas D, Nipkow M, Fiedler G, Schneider R, Haas W, Schürenberg B (2003) Protecting birds from powerlines: a practical guide on the risks to birds from electricity transmission facilities and how to minimise any such adverse effects. In BirdLife International. Report on behalf of the Bern Convention to the Standing Committee of the Convention on the Conservation of European Wildlife and Natural Habitats, 23. Meeting Strasbourg. Available at: <https://www.cms.int/sites/default/files/publication/Protecting_birds_on_powerlines.pdf>

Henkin D, Keitt B (2020) Fixing Hawai'i’s Power Lines and Street Lights to Save Seabirds and Sea Turtles. Earth Justice. <https://earthjustice.org/article/fixing-hawaiis-power-lines-and-street-lights-to-save-seabirds-and-sea-turtles>

Jacobo J (2022) Wildlife could pose 'catastrophic' risks for power grids, experts say. ABC News. Available at: <https://abcnews.go.com/Technology/wildlife-pose-catastrophic-risks-power-grids-experts/story?id=84427141>

Joshi AR (2023) Nepal’s hydropower boom is killing birds via power lines. Mongabay. Available at: <https://news.mongabay.com/2023/05/nepals-hydropower-boom-is-killing-birds-via-power-lines/>

Justo MM, Garrido Lopez RJ, Clavero Sousa H, Barrios V (2022) Wildlife and power lines : guidelines for preventing and mitigating wildlife mortality associated with electricity distribution networks. IUCN. Available at: <https://portals.iucn.org/library/node/50657>

Ketchell M (2023) Roads and power lines put primates in danger: South African data adds to the real picture. The Conversation. Available at: <https://theconversation.com/roads-and-power-lines-put-primates-in-danger-south-african-data-adds-to-the-real-picture-200554>

Kimmel S (2023) The Problem with Power Lines: Electrocution of Sloths in Costa Rica. Cornell University. Available at: <https://wildlife.cornell.edu/blog/problem-power-lines-electrocution-sloths-costa-rica>

Knapton S (2018) Electromagnetic radiation from power lines and phone masts poses 'credible' threat to wildlife, report finds. The Telegraph. Available at: <https://www.telegraph.co.uk/science/2018/05/17/electromagnetic-radiation-power-lines-phone-masts-poses-credible/>

Kovac A (2024) Electrocuted Birds Are Bursting Into Flames and Starting Wildfires. Gizmodo. Available at: <https://gizmodo.com/electrocuted-birds-are-bursting-into-flames-and-starting-wildfires-2000495354>

Krahel O (2023) Power Pylons: Extraordinary Oasis for Wildlife in Farmlands. European Wilderness Society. Available at: <https://wilderness-society.org/power-pylons-extraordinary-oasis-for-wildlife-in-farmlands/>

Kukreti I (2020) NGT wants underground power lines, bird diverters at Great Indian Bustard habitat. Down to Earth. Available at: <https://www.downtoearth.org.in/wildlife-biodiversity/ngt-wants-underground-power-lines-bird-diverters-at-great-indian-bustard-habitat-74759>

Life with Vultures (2022) How dangerous power lines can be made safer for birds. EU Life Project, Natura 2000. Available at: <https://lifewithvultures.eu/how-dangerous-power-lines-can-be-made-safer-for-birds/>

Lundeberg S (2021) Electrical transmission lines have power to enhance habitat connectivity for wildlife. Oregon State University. Available at: <https://news.oregonstate.edu/news/electrical-transmission-lines-have-power-enhance-habitat-connectivity-wildlife>

Macdonald C (2018) Is our addiction to technology putting wildlife at risk? Experts warn electromagnetic radiation from WiFi and power lines poses a 'credible threat' to Earth's species. Daily Mail UK. Available at: <https://www.dailymail.co.uk/sciencetech/article-5746641/Experts-warn-radiation-WiFi-power-lines-poses-credible-threat-Earths-species.html>

Province of Mantioba (2024) Effects of the Physical Presence of Transmission Lines. Available at: <https://www.gov.mb.ca/sd/eal/registries/5750mbhydrombminnesota/cec_docs/ssccecround2_ir397part2.pdf>

Manville II AM (2005) Bird strike and electrocutions at power lines, communication towers, and wind turbines: state of the art and state of the science - next steps toward mitigation. General Technical Report, Pacific Southwest Research Station. In: Ralph, C. John; Rich, Terrell D., editors 2005. Bird Conservation Implementation and Integration in the Americas: Proceedings of the Third International Partners in Flight Conference. 2002 March 20-24; Asilomar, California, Volume 2 Gen. Tech. Rep. PSW-GTR-191. Albany, CA: U.S. Dept. of Agriculture, Forest Service, Pacific Southwest Research Station: p. 1051-1064. Available at: <https://research.fs.usda.gov/treesearch/32105>

Marques AT (2022) Eagles vs. power lines: attraction or avoidance? British Ornithologists Union. Available at: <https://bou.org.uk/blog-marques-eagles-powerlines/>

McCann K (2005) Bird Impact Assessment Study – Braamhoek/Venus 400kV Transmission powerline KwaZulu-Natal, Endangered Wildlife Trust. Available at: <https://www.eskom.co.za/eia/gx/wp-content/uploads/migrated/Ingula-PSS-Access-Roads-And-Transmission-Lines/BVLine_Appendix7_Avifauna.pdf>

McGlashen A (2021) Bird Safety a Concern in National Push to Build More Power Lines. Audubon Society. Available at: <https://www.audubon.org/news/bird-safety-concern-national-push-build-more-power-lines>

McGowan B, Anderson M, Puigcerver L (2020) Increase Reliability in the Power Grid and Reduce Wildlife-Related Fire Risk. Transmission and Distribution (T&D) World. Available at: <https://www.tdworld.com/wildfire/article/21142039/te-connectivity-increase-reliability-in-the-power-grid-and-reduce-wildlife-related-fire-risk>

McKenzie (2015) Nest boxes lessen impact of Jimboomba to Loganlead power line on wildlife. Jimboomba Times. Available at: <https://www.jimboombatimes.com.au/story/3071651/nest-boxes-lessen-impact-of-power-line-on-wildlife/>

Midsun IKM (2023) Bird wingspan versus space between powerlines. Available at: <https://www.midsunikm.com/post/bird-wingspan-versus-space-between-power-lines>

Midsun IKM (2023) Mitigation Measures for Powerline-related bird mortality. Available at: <https://www.midsunikm.com/post/mitigation-measures-for-powerline-related-bird-mortality>

Nally A (2022) Wildlife caused 445 Queensland electricity network outages in 2021, says Energex. ABC News. Available at: <https://www.abc.net.au/news/2022-03-24/queensland-electricity-network-outages-caused-by-wildlife/100931960>

National Board for Wildlife, Ministry of Environment and Forests, India (2011) Guidelines for linear infrastructure intrusions in natural areas: roads and powerlines. Available at: <https://moef.gov.in/uploads/2018/04/FIRSTDraft-guidelines-roads-and-powerlines.pdf>

National Wildlife Federation (NWF) (2023) A Clean Energy Transmission Policy Platform for Thriving Communities and Wildlife. Available at: <https://www.nwf.org/Educational-Resources/Reports/2023/Clean-Energy-Transmission-Policy-Platform>

Okoth D (2024) Africa’s raptors at risk of extinction - Study reveals huge declines in numbers of ecosystem ‘cleaners’. Nature. <https://www.nature.com/articles/d44148-024-00029-6>

Oregon State University (2020) Better way to keep birds from hitting power lines. Available at: <https://www.sciencedaily.com/releases/2020/06/200624151533.htm>

Ottenburghs J (2023) The density of breeding birds increases with distance from electric power lines. British Ornithologist’s Union. Available at: <https://bou.org.uk/blog-jo-avoiding-power-lines/>

Pallet J (2022) Big birds, big power lines, big problems. Conservation Namibia. Available at: <https://conservationnamibia.com/articles/birds-and-power-lines-2022.php>

Peterson C (2023) People are shooting birds off power lines in the West. High Country News. Available at: <https://www.hcn.org/articles/birds-people-are-shooting-birds-off-power-lines-in-the-west/>

Power Africa (2022) Keeping Wildlife Safe and the Lights on Where Nature and Energy Infrastructure Meet in Southern Africa. Medium. Available at: <https://powerafrica.medium.com/keeping-wildlife-safe-and-the-lights-on-where-nature-and-energy-infrastructure-meet-in-southern-ef2b960a60e>

Prinsen HAM, Smallie JJ, Boere GC, Píres N (2012) Guidelines on How to Avoid or Mitigate Impact of Electricity Power Grids on Migratory Birds in the African-Eurasian Region. AEWA Conservation Guidelines No. 14, CMS Technical Series No. 29, AEWA Technical Series No. 50, CMS Raptors MOU Technical Series No. 3, Bonn, Germany. Available at:

<https://www.unep-aewa.org/sites/default/files/publication/ts50_electr_guidelines_03122014.pdf>

Quartucci S (2024) Costa Rica Takes Action to Protect Wildlife from Power Line Hazards. Available at: <https://latinarepublic.com/2024/02/09/costa-rica-takes-action-to-protect-wildlife-from-power-line-hazards/>

Raptor Protection of Slovakia (2023) Electrocutions & Collisions of Birds in EU Countries: The Negative Impact & Best Practices for Mitigation. Available at: <https://www.nabu.de/imperia/md/content/nabude/vogelschutz/stromtod/220628_nabu_studie_electrocutions_and_collisions_of_birds_in_eu-countries.pdf>

Ricciuti E (2019) Fear the Squirrel: How Wildlife Causes Major Power Outages. Cool Green Science – Stories of the Nature Conservancy. Available at: <https://blog.nature.org/2019/10/29/fear-the-squirrel-how-wildlife-causes-major-power-outages/>

Riley L (2023) Finding new ways to work with nature, together. U.S. Fish & Wildlife Service. Available at: <https://www.fws.gov/story/2023-03/power-partnerships>

SA Power Networks (2019) Animals and electricity supply. Available at: <https://www.sapowernetworks.com.au/data/303696/animals-and-electricity-supply/>

Schroeder MA (2010) Greater Sage-grouse and Power Lines: Reasons for Concern. Washington Department of Fish and Wildlife. Available at: <https://wdfw.wa.gov/sites/default/files/publications/01303/wdfw01303.pdf>

Shannon L (2016) Powerlines kill more Tasmanian wedge-tailed eagles than official counts suggest, expert warns. ABC News. Available at: <https://www.abc.net.au/news/2016-05-11/endangered-wedge-tailed-eagle-electrocuted-in-southern-tasmania/7405768>

Sielicki J, Cardenal AC, Conzo LA, Garrido JR, Martin JM, Adamczyk R (2020) Quick Guidance Preventing Electrocution on Birds. International Association for Falconry and Conservation of Birds of Prey (IAF). Available at: <https://birdelectrocution.org/quick-guidance-preventing-electrocution-on-birds/>

Sims J (2021) The Impact of Birds of Prey on Power. Critter Guard. Available at: <https://www.critterguard.org/blogs/articles/the-impact-of-birds-of-prey-on-power>

Shaw J (2016) Guidance - Assessment and mitigation of impacts of power lines and guyed meteorological masts on birds. NatureScot. Available at: <https://www.nature.scot/doc/guidance-assessment-and-mitigation-impacts-power-lines-and-guyed-meteorological-masts-birds>

Shankar Raman TR, Madhusudan MD (2015) Current Ecological Concerns in the Power Sector: Options to Avoid or Minimise Impacts. Socio-Ecological Narratives. Available at: <https://images.assettype.com/ncfindia/import/publications/000/000/373/original/Raman_20and_20Madhusudan_202015_20Current_20Ecological_20Concerns.pdf>

Sloth Conservation Foundation (2024) Power Line Insulation. Available at: <https://slothconservation.org/what-we-do/power-line-insulation/>

Sustainable Development Goals (SDG) Knowledge Hub (2011) UNEP/CMS Reports Highlight Threats to Birds from Power Lines. Available at: <https://sdg.iisd.org/news/unepcms-reports-highlight-threats-to-birds-from-power-lines/>

TasNetworks (2022) Threatened Bird Strategy. Available at: <https://www.tasnetworks.com.au/about-us/corporate-social-responsibility/environment-and-sustainability/report-a-wildlife-incident/tasnetworks-threatened-bird-strategy>

The Habitats Trust (2021) How ‘Green’ Energy Impacts Wildlife. Available at: <https://www.thehabitatstrust.org/features/how-green-energy-impacts-wildlife>

Thiel M (2023) Launch of LIFE SafeLines4Birds project to reduce mortality of birds along power lines. Renewables Grid Initiative. Available at: <https://renewables-grid.eu/publications/press-releases/detail/news/launch-of-life-safelines4birds-project-to-reduce-mortality-of-birds-along-power-lines.html>

TONI – Bird Control Solutions (2024) Bird safety on overhead lines. Available at: <https://www.birdcontrolsolutions.net/en/05_birdprotection/powerlines.php>

University College London (UCL) (2014) Invisible light bursts are keeping animals away from power lines. Available at: <https://www.ucl.ac.uk/news/2014/mar/invisible-light-bursts-are-keeping-animals-away-power-lines>

United Nations (2011) UN review provides guidelines to stop millions of bird deaths due to power lines. Available at: <https://news.un.org/en/story/2011/11/396192-un-review-provides-guidelines-stop-millions-bird-deaths-due-power-lines>

United Nations Environment Programme (2024) 5 surprising obstacles to migratory animals’ journeys. World Conservation Monitoring Centre. Available at: <https://www.unep-wcmc.org/en/news/5-surprising-obstacles-to-migratory-animals-journeys>

Urban Agenda Platform (2017) Improving power distribution in South Africa by addressing wildlife interactions with electrical infrastructure. Dubai International Award. Available at: <https://www.urbanagendaplatform.org/best-practice/improving-power-distribution-south-africa-addressing-wildlife-interactions-electrical>

USAID (2024) - <https://pdf.usaid.gov/pdf_docs/PA00Z9QC.pdf> *Note this reference has expired due to the removal of the U.S. Agency for International Development – 24/01/2025.

U.S. Fish & Wildlife Service (2024) Eagle Incidental Take Permits for Power Lines. Available at: <https://www.fws.gov/program/eagle-management/power-line-permits>

U.S. Fish & Wildlife Service (2024) Electric Transmission Lines. Available at: <https://www.fws.gov/node/266176>

Vieru T (2014) Power Lines Have Worldwide Impact on Wildlife. Available at: <https://news.softpedia.com/news/Power-Lines-Have-Worldwide-Impact-on-Wildlife-432028.shtml>

Voronova V (2011) Assessing of impact of powerlines on birds in Central Kazakhstan steppes. Karaganda Ecological Museum and Association for the Conservation of Biodiversity of Kazakhstan. Available at: <https://www.conservationleadershipprogramme.org/wp-content/uploads/2024/12/0448711_Kazakhstan_FinalReport_Birds.pdf>

Vulture Conservation Foundation (VCF) (2022) Bird collisions: Identifying and securing dangerous power lines to help safeguard the threatened Griffon Vulture in Cyprus. Available at: <https://4vultures.org/blog/bird-collisions-identifying-and-securing-dangerous-power-lines-to-help-safeguard-the-threatened-griffon-vulture-in-cyprus/>

Wagner D, Frye H (2019) New England Power Line Corridors Harbor Rare Bees and Other Wild Things. University of Connecticut. Available at: <https://today.uconn.edu/2019/10/new-england-power-line-corridors-harbor-rare-bees-wild-things/>

Western Power (2024) How we’re keeping WA's threatened bird species safe. Available at: <https://www.westernpower.com.au/about/what-we-do/greening-our-operations/how-were-keeping-was-threatened-bird-species-safe/>

Wildlife Institute of India (2018) Power-Line Mitigation Measures. Available at: <https://wii.gov.in/images/images/documents/publications/rr_2020_GIB%20Power-line_mitigation_conserve_bustards.pdf>

Wildlife Society (2019) Power Line Corridors Support Wildlife. Available at: <https://wildlife.org/power-line-corridors-support-wildlife/>

Williams JH (2003) International Best Practices for Assessing and Reducing the Environmental Impacts of High-Voltage Transmission Lines. Third Workshop on Power Grid Interconnection in Northeast Asia, Vladivostok, Russia, September 30-October 3. Available at: <https://www.nautilus.org/wp-content/uploads/2015/06/Env_Best_Practices_Williams_final.pdf>

Wilke C (2023) Electrocution Isn’t the Main Thing Killing Birds Along Power Lines. The New York Times. Available at: <https://www.nytimes.com/2023/08/03/science/birds-shot-power-lines.html>

WIRES (2024) Flying-Foxes. Available at: <https://www.wires.org.au/wildlife-information/flying-foxes>

Yahoo (2004) Koala 'precariously' hanging from power lines helped by kindhearted locals. Available at: <https://au.news.yahoo.com/koala-precariously-hanging-from-power-lines-helped-by-kindhearted-locals-064257437.html>

Yirka B (2014) Researchers suggest wild animals avoid powerlines due to UV light emittance. Available at: <https://phys.org/news/2014-03-wild-animals-power-lines-due.html>

Yong E (2009) Power lines disrupt the magnetic alignment of cows and deer. National Geographic. Available at: <https://www.nationalgeographic.com/science/article/power-lines-disrupt-the-magnetic-alignment-of-cows-and-deer>
